# Supplementary material for: Design of carbon supports for metal-catalyzed acetylene hydrochlorination
Source: Nat Commun. 2021 Jun 29;12:4016. doi: 10.1038/s41467-021-24330-2 (PMC8242080; doi:10.1038/s41467-021-24330-2)
Supplement: Supplementary file 1 — Supplementary Information [file 41467_2021_24330_MOESM1_ESM.pdf]

Supplementary Information

Design of Carbon Supports for Metal-Catalyzed Acetylene Hydrochlorination

Kaiser *et al*.

Table of Contents

Supplementary Methods 2

Catalyst Preparation 2

Catalyst Characterization 3

Catalytic Evaluation 5

Supplementary Discussions 8

^{13}C Isotope Labeling Study 8

Assessment of Mass and Heat Transfer Limitations 8

Supplementary Tables 11

Supplementary Figures 26

Supplementary References 50

Supplementary Methods

Catalyst Preparation

As summarized in Supplementary Table 1, 11 carbons were either directly employed as commercially available supports or after activation (temperature, $T = 1123$-1153 K, heating rate, $\dot{T} = 5$ K min^{-1}, hold time, $t = 1$-3 h, flowing CO_2) or thermal treatment ($T = 573$-1173 K, $\dot{T} = 5$ K min^{-1}, $t = 1$ h, flowing N_2). The support C2 was prepared via carbonization of olive stone ($T = 1123$ K, $\dot{T} = 5$ K min^{-1}, $t = 2$ h, flowing N_2). CeO_2 was obtained via thermal decomposition ($T = 623$ K, $\dot{T} = 5$ K min^{-1}, $t = 2$ h, static air) of $Ce(NO_3)_3{\cdot}6H_2O$ (Sigma-Aldrich, 99.9%).

^{13}C isotope labeled nitrogen-doped carbon ($N^{13}C$) was prepared via a two-step synthesis, comprising the oxidative polymerization of aniline and a subsequent carbonization step.[1] Accordingly, $^{13}C_6$-aniline (2.7 mmol, Sigma-Aldrich, 99.5%) was dissolved in deionized water (2.15 cm^3, pH 0.4; adjusted by hydrochloric acid, 1.25 M, Sigma-Aldrich, >37%), cooled to 277 K and subsequently added to a precooled solution (277 K) of ammonium persulfate (2.7 mmol, Acros, 98%) in deionized water (1 cm^3) under vigorous stirring, using a magnetic stirring bar. Subsequently, the polyaniline slurry was kept for 24 h at room temperature without stirring, followed by filtering and washing with water (~750 cm^3 g^{-1}), drying ($T = 393$ K, $t = 12$ h, static air), and carbonization ($T = 1073$ K, $\dot{T} = 5$ K min^{-1}, $t = 1$ h, flowing N_2).

All metal-based catalysts were prepared via an incipient wetness impregnation method with a nominal metal loading of 1 wt.%. Accordingly, the metal precursors, H_2PtCl_6 (ABCR, 99.9%), $RuCl_3{\cdot}xH_2O$ (ABCR, 99.9%), and $HAuCl_4{\cdot}xH_2O$ (ABCR, 99.9%), were dissolved in deionized water (for Pt and Ru)[2,3] and acetone (99.5%, for Au)[4] and the obtained solutions were added dropwise to the different carbons and ceria. After impregnation, all carbon-based samples and Pt/CeO_2 were dried (Pt/C: $T = 473$ K, $\dot{T} = 5$ K min^{-1}, $t = 12$ h, static air; CeO_2: $T = 623$ K, $\dot{T} = 5$ K min^{-1}, $t = 1$ h, flowing air).[5] The obtained carbon series, termed Pt/C, Ru/C, and Au/C, exhibit distinct metal nanostructures, as summarized in Supplementary Table 1. Specifically, in the Pt/C and Pt/CeO_2 catalysts, platinum is predominantly present as single atoms, while a few clusters and small nanoparticles were also observed in the case of Pt/C1 and Pt/C2 (Supplementary Fig. 1-5,14). The gold nanostructure strongly depends on the choice of the carbon host and varies from large nanoparticles (Au/C1) to mixed nanostructures (Au/AC3, Au/AC5), and ultimately to single atoms (Au/AC). For Ru/C, small nanoparticles with an average particle

size of 1.5-3.0 nm were obtained, regardless of the type of carbon support Supplementary Fig. 21). Catalysts after use in acetylene hydrochlorination for 12-h time-on-stream (TOS) are labeled metal/C-12h.

The composite Pt/CeO_2+AC catalysts were prepared from a 1:1 weight ratio of Pt/CeO_2 and AC (2 g each) via milling for 30 min at 20 Hz (ball-milled sample) and 5 min at 5 Hz (physical mixture sample) in a Retsch MM 500 nano mixer mill using a 50 cm^3 stainless steel jar and 12 stainless steel balls (0.9 cm diameter, total weight of 48.5 g).

Catalyst Characterization

An overview of the multiple characterization techniques employed in this study and their purposes are summarized in Supplementary Table 2. Powder X-ray diffraction (XRD) was measured using a PANalytical X'Pert PRO-MPD diffractometer with Cu-Kα radiation (λ = 1.54060 Å). The data was recorded in the 10-70° 2θ range with an angular step size of 0.017° and a counting time of 0.26 s per step. Raman spectroscopy was carried out in a confocal Raman microscope (WITec CRM 200) using a 532 nm diode laser. The microscope was operated in the backscattering mode with a 100× objective lens and 4 mW power. N_2 and CO_2 sorption isotherms were measured at 77 K and 273 K, respectively, in a Quantachrome Autosorb-6B equipment after degassing of the solids at 523 K for 4 h. Scanning transmission electron micrographs (STEM) with a high-angle annular dark-field (HAADF) detector were acquired on an aberration-corrected HD2700CS (Hitachi) microscope operated at 200 kV. Samples were prepared by dipping the copper grid supporting a holey carbon foil in a suspension of the solid in ethanol and drying in air. X-ray absorption fine structure (XAFS) measurements at the Pt L_3-edge were carried out at the SuperXAS beamline at the Swiss Light Source (Villigen, Switzerland). The incident photon beam provided by a 2.9 T superbend magnet was selected by a Si(111) channel-cut Quick-EXAFS monochromator.[6] The rejection of higher harmonics and focusing were achieved with rhodium-coated collimating and torroidal mirrors, respectively, at 2.5 mrad. The beamline was calibrated using Pt foil. The area of sample illuminated by the X-ray beam was 0.5 mm×0.2 mm. The Pt/C catalysts (250 mg) were finely ground and mounted in a 3 cm long, 0.5 cm wide cylindrical tube and measured in transmission geometry along the length. All spectra were recorded in transmission mode at room temperature. The extended X-ray absorption fine structure (EXAFS) spectra were acquired with a 1 Hz frequency (0.5 s per spectrum) and then averaged over 5 min. The X-ray

absorption near-edge structure (XANES) spectra were calibrated by measuring Pt foil simultaneously with each sample using the ProQEXAFS software.[7] The XAFS spectra were analyzed using the Demeter software package.[8] The background signal before the Pt L_3-edge was subtracted using a victoreen function (fitting range between –200 and –70 eV). The post edge signal was normalized to the step of one after fitting it in the region between 150 and 1300 eV after the edge. We fitted the k^3 weighted Fourier transformed signal and determined an amplitude reduction factor (S_0^2) of 0.84 from EXAFS fit of the Pt metal foil. All EXAFS spectra were fitted for the first coordination shell in the k-range of 3-16.8 Å^{-1} and R-range of 1-3 Å. To fit the Pt-Pt, Pt-Cl and Pt-(O/C) scattering paths, Pt foil, H_2PtCl_6 and PtO_2 were used as the references. X-ray photoelectron spectra (XPS) were acquired on a Physical Electronics Quantera SXI instrument using monochromatic Al-Kα radiation, generated from an electron beam operated at 15 kV, and equipped with a hemispherical capacitor electron-energy analyzer. The samples were analyzed at an electron take-off angle of 45° and a constant analyzer pass energy of 50.0 eV with a spectra resolution step width of 0.1 eV. To suppress sample charging during analysis, an electron and an ion neutralizer were operated simultaneously. The spectrometer was calibrated for the Au 4$f_{7/2}$ signal at 84.0 ± 0.1 eV. The spectra were fitted by mixed Gaussian-Lorentzian component profiles after Shirley background subtraction. The Pt 4f signal was fitted with two components assigned to Pt IV (73.5 ± 0.2 eV) and Pt II (72.4 ± 0.1 eV). The $Pt^{7/2}$ and $Pt^{5/2}$ doublet was constrained to be separated by 3.3 eV and at an areal ratio of 4:3.[2] Two contributions assigned to C-Cl (200.0 ± 0.1 eV) and Pt-Cl (198.1 ± 0.2 eV) were fitted to the Cl 2p signal constraining the $Cl^{3/2}$ and $Cl^{1/2}$ doublet to an areal ratio of 2:1 separated by 1.6 eV.[9] The O 1s signals were fitted using three components assigned to adsorbed water (536.5-536.0 eV), C-O (532.8 ± 0.1 eV), and C=O (531.1 ± 0.1 eV).[10] The Ru 3$p_{3/2}$ signal was fitted with three components assigned to metallic Ru(0) (461.25 ± 0.15 eV), RuO_2 (462.75 ± 0.15 eV), and $RuCl_3$ (464.25 ± 0.15 eV).[11] The surface chemistry of the carbon supports was characterized with a SDT 2960 thermobalance from TA Instruments coupled to a Balzers GSD 300 T3 Thermostar mass spectrometer (TGA-MS). Accordingly, the sample (0.1 g) was heated up (T = 1200 K, $\dot{T}$ = 10 K min^{-1}, total volumetric flow, F_T = 100 cm^3 min^{-1}, flowing He) and the evolved products (CO and CO_2) were analyzed by MS. Temperature-programmed desorption analyses of C_2H_2, HCl, C_2H_3Cl, and NH_3 were performed in a Micromeritics Autochem II 2920 analyzer equipped with a thermal conductivity detector (TCD) and a Pfeiffer Vacuum OmniStar GSD 320 O mass spectrometer (TPD-MS). The

samples (0.1 g) were loaded into a U-shaped quartz micro-reactor, pre-dried (T = 533 K, $\dot{T}$ = 10 K min^{-1}, t = 20 min, F_T = 20 cm^3 min^{-1}, flowing He), cooled to 303 K, and subsequently saturated with a flow of the respective probe molecule (T = 303 K, t = 20 min, F_T = 20 cm^3 min^{-1}). After purging (T = 303 K, t = 20 min, F_T = 20 cm^3 min^{-1}, flowing He), the desorption was initiated by increasing the temperature (T = 533 K, $\dot{T}$ = 5 K min^{-1}, flowing He) while monitoring the desorbed products by MS. To quantify the fraction of acetylene desorbing at temperatures >473 K (reaction temperature), we integrated (i) the whole data range between 303 K and 533 K (indicated as total acetylene adsorption capacity) and (ii) the fraction of the data range between 473 K and 533 K (referred to as acetylene adsorption capacity above 473 K). To assess the impact of HCl treatment on the acetylene adsorption capacity, the samples (0.1 g) were dried (T = 533 K, $\dot{T}$ = 10 K min^{-1}, t = 20 min, F_T = 20 cm^3 min^{-1}, flowing He), pre-treated (T = 473 K, t = 30 min, F_T = 15 cm^3 min^{-1}, flowing HCl) and cooled to 303 K, prior to TPD-MS analysis. Volumetric chemisorption of acetylene was performed in a Micromeritics 3Flex Chemi instrument at 303 K and 473 K to quantify the amount of acetylene chemi (V_{chem}) and physisorbed (V_{phys}) at the respective temperatures. To quantify the amount of coke in the used catalysts thermogravimetric analysis, TGA, of the fresh and used catalysts (10 mg) was performed using a Linseis STA PT1600 thermobalance coupled to a Pfeiffer Vacuum Thermo-Star GDS 320 T1 mass spectrometer (TGA-MS, T = 1073 K, $\dot{T}$ = 10 K min^{-1}, F_T = 100 cm^3 min^{-1}, flowing 20 vol.% O_2/Ar).

Catalytic Evaluation

The hydrochlorination of acetylene was evaluated at atmospheric pressure in a continuous-flow fixed-bed micro-reactor (Supplementary Fig. 24). The gases C_2H_2 (PanGas, purity 2.6), HCl (Air Liquide, purity 2.8, anhydrous), Ar (PanGas, purity 5.0, internal standard), and He (PanGas, purity 5.0, carrier gas), were fed using digital mass-flow controllers (Bronkhorst) to the mixing unit, equipped with a pressure indicator. A quartz micro-reactor of 10 mm inner diameter containing a porous frit was loaded with the catalyst (W_{cat} = 0.1 g for initial catalytic activity tests and kinetic tests, and 0.1-0.25 g for stability tests) and placed in a homemade electrical oven. A K-type thermocouple fixed in a coaxial quartz thermowell with the tip positioned in the center of the catalyst bed was used to control the temperature during the reaction. Prior to catalytic tests, the catalyst was heated (bed temperature, T_{bed} = 473 K, t = 30 min, flowing He). Thereafter, a total gas flow, F_T = 15 cm^3 min^{-1}, containing 40 vol.% C_2H_2, 44 vol.% HCl, and 16 vol.% Ar, was fed into the reactor at bed temperatures, T_{bed} = 453-483 K, employing a high gas hourly space velocity

based on acetylene, $GHSV(C_2H_2) = 650\text{-}1500\ h^{-1}$ to assess the catalysts under accelerated deactivation conditions. Kinetic studies of acetylene hydrochlorination over Pt/C catalysts were conducted (T_{bed} = 453-483 K, F_T = 20 cm^3 min^{-1}, 10-30 vol.% C_2H_2 and HCl in He) to determine the apparent activation energy (E_a) and the partial reaction order of the reactants ($n_{C_2H_2}$, n_{HCl}). Carbon-containing compounds (C_2H_2 and C_2H_3Cl) and Ar were quantified on-line via a gas chromatograph equipped with a GS-Carbon PLOT column coupled to a mass spectrometer (GC-MS, Agilent, GC 7890B, Agilent MSD 5977A). For the ^{13}C isotope labeling study, the reactor outlet was directly connected to a Pfeiffer Vacuum Thermo-Star GDS 320 T1 MS. Since vinyl chloride (VCM) was the only product detected in all our tests, the catalytic activity is presented as the yield of VCM, Y(VCM), calculated according to Eq. 1,

$$Y(\text{VCM}),\% = \frac{n_{\text{VCM}}^{\text{outlet}}}{n_{\text{C}_2\text{H}_2}^{\text{inlet}}} \cdot 100 \quad (1)$$

where $n_{\text{VCM}}^{\text{outlet}}$ and $n_{\text{C}_2\text{H}_2}^{\text{inlet}}$ denote the molar flows of VCM and C_2H_2 at the reactor outlet and inlet, respectively. The reaction rate (r), turnover frequency (TOF), and space-time-yield (STY) were determined according to Eq. 2-4,

$$r, \text{mol}_{\text{C}_2\text{H}_2}\ \text{s}^{-1}\ \text{g}_{\text{cat}}{}^{-1} = \frac{n_{\text{C}_2\text{H}_2}^{\text{inlet}} - n_{\text{C}_2\text{H}_2}^{\text{outlet}}}{W_{\text{cat}}} \quad (2)$$

$$\text{TOF}, \text{mol}_{\text{C}_2\text{H}_2}\ \text{s}^{-1}\ \text{mol}_{\text{metal}}{}^{-1} = \frac{n_{\text{C}_2\text{H}_2}^{\text{inlet}} - n_{\text{C}_2\text{H}_2}^{\text{outlet}}}{n_{\text{metal}}} \quad (3)$$

$$\text{STY}, \text{g}_{\text{VCM}}\ \text{h}^{-1}\ \text{g}_{\text{cat}}{}^{-1} = \frac{n_{\text{VCM}}^{\text{outlet}}}{W_{\text{cat}}} \cdot M_{\text{VCM}} \quad (4)$$

where W_{cat} denotes the catalyst mass, n_{metal} the mole of metals, and M_{VCM} the molecular weight of VCM. The error of the carbon balance, ε_{C}, determined using Eq. 5, was less than 5% in all experiments, i.e., the carbon mass balance was closed at ≥ 95%.

$$\varepsilon_{\text{C}}, \% = \frac{n_{\text{C}_2\text{H}_2}^{\text{inlet}} - \left(n_{\text{C}_2\text{H}_2}^{\text{outlet}} + n_{\text{VCM}}^{\text{outlet}}\right)}{n_{\text{C}_2\text{H}_2}^{\text{inlet}}} \cdot 100 \quad (5)$$

After the tests, the reactor was quenched to room temperature in He flow and the catalyst was retrieved for further characterization. All catalytic data points were determined as an average of at least three measurements. The evaluation of the dimensionless moduli based on the criteria of Carberry,[12] Mears,[13] and Weisz-Prater,[14] confirmed that all the catalytic tests were performed in

the absence of mass and heat transfer limitations (see for details Supplementary Discussion and Supplementary Table 15). The deactivation constants, k_D, were derived via a simple linear regression of the data range within the first 3 and 12 h time-on-stream in acetylene hydrochlorination ($k_{D,3h}$ and $k_{D,12h}$, Supplementary Table 13).

Supplementary Discussions

^{13}C Isotope Labeling Study

To assess whether carbon could be directly involved in the catalytic cycle of acetylene hydrochlorination (e.g., via a Mars-van Krevelen mechanism), a metal-free nitrogen-doped carbon catalyst[1] was synthesized using ^{13}C labeled aniline (N^{13}C) and tested under typical operating conditions (Supplementary Fig. 20, W_{cat} = 0.1 g, T_{bed} = 473 K, F_T = 15 cm^3 min^{-1}, HCl:C_2H_2 = 1.1:1, GHSV(C_2H_2) = 1500 h^{-1}). Time-resolved MS analysis of the reaction products at the reactor outlet was performed to monitor the relative concentration of produced vinyl chloride (C_2H_3Cl) with *m*:*z* 62, *m*:*z* 63, and *m*:*z* 64. In line with the natural isotopic abundance of ^{35}Cl and ^{37}Cl, *m*:*z* 62 and *m*:*z* 64 were detected in a characteristic 3:1 ratio, indicating non-isotope labeled vinyl chloride. The formation of $^{13}CH_2$-CHCl, CH_2-^{13}CHCl, and $^{13}CH_2$-^{13}CHCl could be excluded, as *m*:*z* 63 and *m*:*z* 64 were not detected in relevant or enriched amounts, respectively. This observation indicates that no carbon-atom exchange takes place during the catalytic cycle. Hence, the unique suitability of carbon for this application cannot be attributed to the role of serving as a source of carbon atoms for the product formation.

Assessment of Mass and Heat Transfer Limitations

The Carberry criterion (Ca)[4] was used to evaluate external mass transfer limitations according to Eq. 6,

$$\mathrm{Ca} = \frac{r_{\mathrm{v,obs}}}{a' \cdot k_{\mathrm{f}} \cdot c_{\mathrm{b}}} < \frac{0.05}{|n|} \qquad \text{Eq. 6}$$

where k_{f} is the mass transfer coefficient (estimated at a minimum value of 0.01 m s^{-1}), c_{b} is the bulk concentration of acetylene (17.6 $\mathrm{mol}_{C_2H_2}$ m^{-3}), n the reaction order, and $r_{\mathrm{v,obs}}$ and a' denote the reaction rate and the specific particle area, which are derived via Eq 7 and Eq. 8, respectively,

$$r_{\mathrm{v,obs}} = \frac{n_{C_2H_2}^{\mathrm{inlet}} - n_{C_2H_2}^{\mathrm{outlet}}}{V_{\mathrm{cat}}} \qquad \text{Eq. 7}$$

$$a' = \frac{1}{L} = \frac{A_{\mathrm{p}}}{V_{\mathrm{p}}} = \frac{6}{d_{\mathrm{p}}} \qquad \text{Eq. 8}$$

To assess external temperature differences (ΔT_{e}),[4] Eq. 9 was applied,

$$\Delta T_{\mathrm{e}} = \beta_{\mathrm{e}} \cdot Ca = \frac{\left(-\Delta H_{\mathrm{r}}\right) \cdot k_{\mathrm{f}} \cdot c_{\mathrm{b}}}{h \cdot T_{\mathrm{b}}} Ca \qquad \text{Eq. 9}$$

where β_{e} denotes the external Prater number, T_{b} the temperature in the bulk material (473 K), h the heat transfer coefficient (estimated at a minimum value of 10 J m^{-2} s^{-1} K^{-1}), and ΔH_{r} the reaction enthalpy (99.3 kJ mol^{-1}). Internal mass transfer limitations were evaluated using the Weisz-Prater criterion (Φ),[5] according to Eq. 10,

$$\Phi = \frac{r_{\mathrm{v,obs}} \cdot L^2}{D_{\mathrm{eff}} \cdot c_{\mathrm{s}}} \left(\frac{n+1}{2}\right) < 1 \qquad \text{Eq. 10}$$

Where L is the characteristic length (0.25 mm), c_{s} the surface concentration ($c_{\mathrm{s}} \approx c_{\mathrm{b}}$ in the absence of external mass transfer limitations), and D_{eff} the effective diffusion coefficient, which can be derived via Eq. 11,

$$D_{\mathrm{eff}} = \frac{\varepsilon}{\tau} \cdot \overline{D} = \left(\frac{1}{D_{\mathrm{C_2H_2,HCl}}} + \frac{1}{D_{\mathrm{K}}}\right)^{-1} \qquad \text{Eq. 11}$$

where τ is the tortuosity factor (estimated at 3), ε is the particle porosity (estimated at 0.2), $D_{\mathrm{C_2H_2,HCl}}$ is the molecular diffusion coefficient (1.96 10^{-5} m^2 s^{-1}), and D_{K} is the Knudsen diffusion coefficient which is calculated for components i (i = HCl, C_2H_2) in a cylindrical pore according to Eq. 12,

$$D_{\mathrm{K},i} = 97 \cdot r_{\mathrm{pore}} \cdot \sqrt{\frac{T}{M_i}} \qquad \text{Eq. 12}$$

where r_{pore} denotes the pore radius, M_{i} the molecular weight of component i, and T the temperature (473 K). Intra-particle temperature differences (ΔT_{i}) can be calculated using Eq. 13,[6]

$$\Delta T_{\mathrm{i}} = \beta_{\mathrm{i}} \cdot T_{\mathrm{s}} = \frac{\left(-\Delta H_{\mathrm{r}}\right) \cdot D_{\mathrm{eff}} \cdot c_{\mathrm{s}}}{\lambda_{\mathrm{eff}}} \qquad \text{Eq. 13}$$

where β_{i} denotes the internal Prater number and λ_{eff} the effective thermal conductivity (estimated at 0.5 W K^{-1} min^{-1}). The results for Pt/C catalysts are given in Supplementary Table 15. For all catalytic tests, the Carberry criterion (Eq. 6) is fulfilled and hence no external mass transfer limitations are present. In line with this result, the external temperature difference is neglectable (< 0.01 K), indicating the absence of external heat transfer limitations. In the fresh Pt/C catalysts, intra-particle mass transfer limitations are largely neglectable, while with decreasing pore size minor diffusional disguises might develop for Pt/AC1 and Pt/AC2, which are partially

compensated by the simultaneous drop in the reaction rate. Only for pore sizes < 0.5 nm internal mass transfer limitations become dominant. Finally, the temperature gradient within the catalyst particle is negligible for each case, indicating the absence of internal heat transfer limitations.

Supplementary Tables

Supplementary Table 1. Catalyst notation, details on the carbon supports employed, and metal speciation.

Catalyst	Support	Company	T[b,c] [K]	t [h]	Atmosphere	Speciation[d]
Pt/AC	ROX-08	Norit	-	-	-	SA
Pt/C1	GE	Gun Ei Chemical Ind.	-	-	-	SA[e]
Pt/AC1	GE	-	1153[b]	3	CO_2	SA
Pt/C2	[a]	[a]	1123[b]	2	N_2	SA[e]
Pt/AC2	C2	-	1123[b]	1	CO_2	SA[e]
Pt/AC3	ACGE	Gun Ei Chemical Ind.	-	-	-	SA
Pt/AC4	WV-1100	Westvaco	-	-	-	SA
Pt/AC4-573	WV-1100	-	573[c]	1	N_2	SA
Pt/AC4-873	WV-1100	-	873[c]	1	N_2	SA
Pt/AC4-1173	WV-1100	-	1173[c]	1	N_2	SA
Pt/AC5	BAC	Kureha corp.	-	-	-	SA
Pt/CeO_2	CeO_2	-	623	2	Air	SA
Ru/C1	GE	Gun Ei Chemical Ind.	-	-	-	NP
Ru/AC	ROX-08	Norit	-	-	-	NP
Ru/AC4	WV-1100	Westvaco	-	-	-	NP
Ru/AC5	BAC	Kureha corp.	-	-	-	NP
Au/C1	GE	Gun Ei Chemical Ind.	-	-	-	NP
Au/AC	ROX-08	Norit	-	-	-	SA
Au/AC3	ACGE	Gun Ei Chemical Ind.	-	-	-	SA+NP[f]
Au/AC5	BAC	Kureha corp.	-	-	-	SA+NP[f]

[a]Char prepared as described in the Catalyst Preparation. [b]Activation temperature. [c]Thermal treatment temperature. [d]Metal speciation: SA: single atoms, NP: nanoparticles. [e]Occasional clusters and small nanoparticles observed. [f]Both NP and SA present.

Supplementary Table 2. Overview of employed techniques, their acronyms, and uses.

Technique (acronym)	Purposes
X-ray diffraction (XRD)	Carbon crystallinity and metal nuclearity
Raman	Structure of carbon
N_2 sorption at 77 K and CO_2 sorption at 273 K	Porous properties
Scanning transmission electron microscopy (STEM) with high-angle annular dark-field (HAADF) detector	Metal nuclearity and dispersion
X-ray absorption fine structure (XAFS) analysis at the Pt L_3-egde	Metal coordination environment
X-ray photoelectron spectroscopy (XPS) of Pt 4*f*, O 1*s*, and Cl 2*p* core level	Surface composition and elemental speciation
Thermogravimetric analysis coupled to mass spectrometry (TGA-MS)	Surface oxygen content and distribution of oxygen functionalities and coke quantification
Temperature-programmed desorption of C_2H_2, HCl, C_2H_3Cl, and NH_3 coupled to mass spectrometry (TPD-MS)	Capacity and strength of interaction with reactants and product as well as amount of acidic sites
Volumetric chemisorption of C_2H_2 at 303 K and 473 K	Quantification of acetylene adsorption capacity and strength as a function of temperature

Supplementary Table 3. EXAFS fitting parameters of fresh and used Pt/C catalysts.

Catalyst	Coordination	CN[a] / -	σ^{2b} / Å^2	R^c / Å
Pt/AC	Pt-C	0.4 ± 0.3	0.006 ± 0.002	1.86 ± 0.04
	Pt-O	0.8 ± 0.3	0.006 ± 0.002	2.10 ± 0.02
	Pt-Cl	3.3 ± 0.3	0.003 ± 0.001	2.31 ± 0.02
	Pt-Cl	0.3[d]	0.003[e]	2.90 ± 0.03
Pt/AC-12h	Pt-C	0.8 ± 0.3	0.004 ± 0.001	1.85 ± 0.02
	Pt-O	0.9 ± 0.3	0.006 ± 0.002	2.10 ± 0.02
	Pt-Cl	2.8 ± 0.2	0.003 ± 0.001	2.32 ± 0.02
	Pt-Cl	0.6[d]	0.003[e]	2.91 ± 0.03
Pt/C1	Pt-O	1.5 ± 0.2	0.006 ± 0.002	2.07 ± 0.02
	Pt-Cl	3.1 ± 0.3	0.003 ± 0.001	2.30 ± 0.02
Pt/C1-12h	Pt-O	1.3 ± 0.2	0.006 ± 0.002	2.10 ± 0.03
	Pt-Cl	1.4 ± 0.2	0.003 ± 0.001	2.32 ± 0.02
	Pt-Pt	1.7 ± 0.6	0.009 ± 0.004	2.77 ± 0.03
Pt/AC3	Pt-O	0.9 ± 0.3	0.006 ± 0.002	2.05 ± 0.02
	Pt-Cl	2.6 ± 0.3	0.003 ± 0.001	2.31 ± 0.02
	Pt-Cl	0.2[d]	0.003[e]	2.91 ± 0.04
Pt/AC3-12h	Pt-C	1.2 ± 0.2	0.005 ± 0.002	1.84 ± 0.03
	Pt-O	0.5 ± 0.3	0.006 ± 0.002	2.11 ± 0.02
	Pt-Cl	3.0 ± 0.3	0.003 ± 0.001	2.31 ± 0.02
	Pt-Cl	0.8[d]	0.003[e]	2.90 ± 0.03
Pt/AC4	Pt-C	0.4 ± 0.2	0.005 ± 0.002	1.88 ± 0.03
	Pt-O	1.2 ± 0.2	0.006 ± 0.002	2.05 ± 0.03
	Pt-Cl	2.1 ± 0.2	0.003 ± 0.001	2.32 ± 0.02
Pt/AC4-12h	Pt-C	0.7 ± 0.4	0.004 ± 0.001	1.84 ± 0.03
	Pt-O	0.3 ± 0.3	0.006 ± 0.002	2.07 ± 0.03
	Pt-Cl	2.9 ± 0.3	0.003 ± 0.001	2.31 ± 0.02
	Pt-Cl	0.4[d]	0.003[e]	2.92 ± 0.04
Pt/AC5	Pt-C	0.7 ± 0.3	0.006 ± 0.002	1.87 ± 0.02
	Pt-O	0.6 ± 0.3	0.006 ± 0.002	2.10 ± 0.03
	Pt-Cl	3.0 ± 0.3	0.003 ± 0.001	2.32 ± 0.02
	Pt-Cl	0.3[d]	0.003[e]	2.91 ± 0.03
Pt/AC5-12h	Pt-C	1.0 ± 0.3	0.006 ± 0.002	1.85 ± 0.02
	Pt-O	0.4 ± 0.2	0.006 ± 0.002	2.12 ± 0.03
	Pt-Cl	3.1 ± 0.3	0.003 ± 0.001	2.31 ± 0.02
	Pt-Cl	0.9[d]	0.003[e]	2.91 ± 0.02

[a]Coordination number. [b]Debye-Waller factor. [c]Coordination shell distance. [d]No uncertainty given due to highly correlating parameters and significant uncertainty in the related Debye waller factor. [e]Debye waller factor held at the refined value for the stronger Pt-Cl coordination at ~2.31 Å due to highly correlating parameters.

Supplementary Table 4. Elemental surface concentrations determined by XPS of fresh and used Pt/C catalysts.

Catalyst	C [at.%]	O [at.%]	Cl [at.%]
Pt/AC	96.6	3.0	0.3
Pt/AC-12h	96.0	2.7	0.8
Pt/C1	93.5	4.8	0.5
Pt/C1-12h	94.9	4.2	0.9
Pt/AC3	94.3	5.2	0.2
Pt/AC3-12h	93.9	4.9	0.5
Pt/AC4	91.8	7.4	0.1
Pt/AC4-12h	92.7	6.5	0.8
Pt/AC4-573	92.5	7.2	0.3
Pt/AC4-573-12h	92.0	6.7	0.5
Pt/AC4-873	93.0	7.0	0.1
Pt/AC4-873-12h	93.8	5.5	0.3
Pt/AC4-1173	93.8	5.7	0.1
Pt/AC4-1173-12h	95.0	4.7	0.3
Pt/AC5	95.3	4.4	0.2
Pt/AC5-12h	95.9	3.3	0.7

Supplementary Table 5. Fitting parameters derived from the Pt 4*f* XPS spectra of fresh and used Pt/C catalysts and Pt/CeO_2.

Catalyst	Pt(IV)[a]			Pt(II)[a]		
	Position [eV]	FWHM[b] [eV]	Area [%]	Position [eV]	FWHM[b] [eV]	Area [%]
Pt/AC	73.6	2.5	17	72.3	2.0	83
Pt/AC-12h	73.5	2.2	93	72.3	2.0	7
Pt/C1	73.3	1.8	18	72.5	1.7	82
Pt/C1-12h	73.5	1.8	31	72.5	1.8	69
Pt/AC3	73.4	2.2	47	72.3	1.4	53
Pt/AC3-12h	73.4	1.8	88	72.3	1.3	12
Pt/AC4	73.3	1.9	49	72.3	1.4	51
Pt/AC4-12h	73.3	1.4	15	72.3	2.0	85
Pt/AC4-573	73.5	1.9	30	72.3	1.7	70
Pt/AC4-573-12h	73.5	1.3	24	72.3	2.4	76
Pt/AC4-873	73.4	1.9	55	72.4	1.5	45
Pt/AC4-873-12h	73.4	1.8	63	72.5	1.9	37
Pt/AC4-1173	73.3	1.9	43	72.5	1.5	57
Pt/AC4-1173-12h	73.4	2.0	68	72.5	2.3	32
Pt/AC5	73.6	2.2	34	72.3	1.7	66
Pt/AC5-12h	73.4	1.6	95	72.3	1.4	5
Pt/CeO_2	73.5	1.3	66	72.5	1.3	34

[a]Assigned based on reference values.[2,15,16] [b]FWHM: full width at half maximum.

Supplementary Table 6. Fitting parameters derived from the Cl 2*p* XPS spectra of fresh and used Pt/C catalysts.

Catalyst	C-Cl[a]			Pt-Cl[a]		
	Position [eV]	FWHM[b] [eV]	Area [%]	Position [eV]	FWHM[b] [eV]	Area [%]
Pt/AC	200.1	2.1	45	197.9	2.0	55
Pt/AC-12h	200.1	1.8	96	198.1	0.6	4
Pt/C1	199.9	1.8	37	198.3	1.8	63
Pt/C1-12h	200.1	1.3	68	197.9	1.9	32
Pt/AC3	200.1	2.1	44	197.9	1.4	56
Pt/AC3-12h	200.1	1.6	74	197.9	1.1	27
Pt/AC4	200.1	1.3	27	198.1	1.4	73
Pt/AC4-12h	200.1	1.8	76	198.3	1.5	24
Pt/AC4-573	200.1	0.8	20	197.9	1.4	80
Pt/AC4-573-12h	199.9	1.5	39	198.0	1.8	61
Pt/AC4-873	200.1	1.3	61	198.1	1.3	39
Pt/AC4-873-12h	200.0	1.5	70	198.1	1.6	30
Pt/AC4-1173	200.1	1.1	17	198.2	1.4	83
Pt/AC4-1173-12h	200.1	1.4	59	198.1	1.4	41
Pt/AC5	200.1	1.4	44	197.9	1.6	56
Pt/AC5-12h	200.1	1.5	84	197.9	1.0	16

[a]Assigned based on reference values.[9] [b]FWHM: full width at half maximum.

Supplementary Table 7. Porous properties of the fresh and used carbon supports and Pt/C catalysts.

Sample	Support					Fresh Pt/C catalyst					Used Pt/C catalyst, TOS = 12 h				
	V_{total}[a] [cm^3 g^{-1}]	$V_{\mathrm{meso,N_2}}$[b] [cm^3 g^{-1}]	$V_{\mathrm{micro,N_2}}$[c] [cm^3 g^{-1}]	$V_{\mathrm{micro,CO_2}}$[d] [cm^3 g^{-1}]	S_{BET}[e] [m^2 g^{-1}]	V_{total}[a] [cm^3 g^{-1}]	$V_{\mathrm{meso,N_2}}$[b] [cm^3 g^{-1}]	$V_{\mathrm{micro,N_2}}$[c] [cm^3 g^{-1}]	$V_{\mathrm{micro,CO_2}}$[d] [cm^3 g^{-1}]	S_{BET}[e] [m^2 g^{-1}]	V_{total}[a] [cm^3 g^{-1}]	$V_{\mathrm{meso,N_2}}$[b] [cm^3 g^{-1}]	$V_{\mathrm{micro,N_2}}$[c] [cm^3 g^{-1}]	$V_{\mathrm{micro,CO_2}}$[d] [cm^3 g^{-1}]	S_{BET}[e] [m^2 g^{-1}]
AC	0.69 (0.67)[g]	0.23 (0.09)[g]	0.44 (0.57)[g]	0.37 (0.43)[g]	901 (1193)[g]	0.58	0.17	0.38	0.34	806	0.39	0.17	0.20	0.20	434
C1	0.10	0.03	0.06	0.13	155	0.04	0.02	0.02	0.22	47	-	-	-	0.20	-
AC1	0.28	0.03	0.24	0.28	529	0.28	0.03	0.26	0.27	512	0.02	0.01	0.01	0.21	-
C2	0.16	0.05	0.12	0.22	248	0.15	0.02	0.12	0.22	255	0.01	0.00	0.00	0.20	4
AC2	0.32	0.03	0.29	0.29	624	0.32	0.03	0.28	0.29	576	0.08	0.02	0.05	0.17	98
AC3	1.05 (0.96)[g]	0.04 (0.04)[g]	0.86 (0.81)[g]	0.62 (0.50)[g]	2113 (1920)[g]	0.98	0.03	0.77	0.52	1924	0.78	0.02	0.69	0.41	1554
AC4	1.21 (1.09)[g]	0.28 (0.24)[g]	0.74 (0.67)[g]	0.39 (0.35)[g]	1800 (1638)[g]	1.09	0.24	0.67	0.36	1637	0.73	0.19	0.39	0.20	1019
573[f]	1.18	0.35	0.70	0.34	1713	1.11	0.22	0.68	0.36	1640	0.98	0.24	0.57	0.29	1412
873[f]	1.11	0.28	0.68	0.34	1642	1.01	0.19	0.62	0.33	1562	0.94	0.19	0.54	0.29	1386
1173[f]	0.85	0.21	0.55	0.33	1320	0.81	0.16	0.50	0.33	1255	0.66	0.15	0.38	0.25	981
AC5	0.59 (0.56)[g]	0.03 (0.04)[g]	0.57 (0.53)[g]	0.42 (0.42)[g]	1159 (1061)[g]	0.57	0.03	0.54	0.43	1097	0.53	0.04	0.53	0.37	938

[a]Volume of N_2 adsorbed at p/p_0 = 0.99 and 77 K. [b]Estimation of volume of mesopores from N_2 adsorption between p/p_0 = 0.9 and 0.2 at 77 K. [c]Total volume of micropores from N_2 adsorption at 77 K. [d]Volume of narrow micropores from CO_2 adsorption at 273 K. [e]Total surface area, BET method. Determination of these porous parameters is based on references.[17-20] [f]AC4 after thermal treatment at indicated temperatures in K. [g]Used support, TOS = 12 h.

Supplementary Table 8. Amount of CO_2, CO, and total oxygen (O_{total}) released from the fresh and used carbon supports and Pt/C catalysts during TGA-MS in flowing He.

Sample	Support			Fresh Pt/C catalyst			Used Pt/C catalyst, TOS = 12 h		
	CO_2 [μmol g^{-1}]	CO [μmol g^{-1}]	O_{total} [μmol g^{-1}]	CO_2 [μmol g^{-1}]	CO [μmol g^{-1}]	O_{total} [μmol g^{-1}]	CO_2 [μmol g^{-1}]	CO [μmol g^{-1}]	O_{total} [μmol g^{-1}]
AC	357 (157)[a]	751 (501)[a]	1465 (815)[a]	343	1465	2151	218	1152	1587
C1	336	417	1089	444	1123	2012	292	936	1519
AC1	152	386	690	334	1064	1732	226	624	1076
C2	434	357	1225	381	953	1715	280	935	1494
AC2	142	273	556	185	776	1146	207	604	1019
AC3	224 (156)[a]	1787 (1547)[a]	2236 (1859)[a]	269	1826	2365	175	1407	1757
AC4	540 (452)[a]	2622 (2569)[a]	3702 (3473)[a]	726	3463	4915	474	2662	3610
AC4-573	356	2569	3281	605	3415	4624	511	3123	4145
AC4-873	166	1574	1906	422	2894	3739	355	3005	3715
AC4-1173	198	985	1380	380	2029	2788	244	1907	2395
AC5	105 (72)[a]	239(298)[a]	449 (442)[a]	138	881	1157	77	951	1105

[a]Used support, TOS = 12 h.

Supplementary Table 9. Fitting parameters derived from the O 1*s* XPS spectra of fresh and used Pt/C catalysts.

Catalyst	H_2O_{ads}[a]			C-O[a]			C=O[a]		
	Position [eV]	FWHM[b] [eV]	Area [%]	Position [eV]	FWHM[b] [eV]	Area [%]	Position [eV]	FWHM[b] [eV]	Area [%]
Pt/AC	536.5	2.5	12	532.9	2.5	70	531.2	1.6	18
Pt/AC-12h	536.5	2.5	17	532.9	2.5	83	531.1	1.7	0
Pt/C1	536.5	1.0	0	532.7	2.5	83	531.2	1.6	17
Pt/C1-12h	536.3	1.2	1	532.7	2.0	68	531.2	1.7	31
Pt/AC3	536.5	2.5	3	532.9	2.5	71	531.1	1.9	26
Pt/AC3-12h	536.5	2.5	6	532.9	2.5	78	531.1	1.7	16
Pt/AC4	536.4	2.5	5	532.9	2.5	63	531.0	1.8	31
Pt/AC4-12h	536.2	2.5	8	532.9	2.5	71	531.1	1.6	22
Pt/AC4-573	536.3	2.5	8	532.9	2.4	54	531.0	2.0	38
Pt/AC4-573-12h	536.2	2.5	9	532.9	2.2	43	531.0	2.5	48
Pt/AC4-873	536.5	2.1	4	532.9	2.4	71	531.1	1.5	25
Pt/AC4-873-12h	536.3	2.3	8	532.9	2.3	60	531.0	1.8	33
Pt/AC4-1173	536.2	2.5	8	532.8	2.5	67	531.0	1.9	25
Pt/AC4-1173-12h	536.0	2.1	5	532.8	2.4	70	531.0	2.0	25
Pt/AC5	536.5	2.5	7	532.9	2.4	73	531.1	1.7	20
Pt/AC5-12h	536.5	2.5	10	532.9	2.5	73	531.2	1.8	17

[a]Assigned based on reference values.[10,21] [b]FWHM: full width at half maximum.

Supplementary Table 10. Adsorption capacities of ammonia, acetylene, hydrogen chloride, and vinyl chloride of fresh Pt/C catalysts expressed as the integral of the corresponding MS signal monitored during TPD-MS.

Catalyst	NH_3 [a.u.]	C_2H_2 [a.u.]	HCl [a.u.]	VCM [a.u.]
Pt/AC	1.59	2.14	-	0.42
Pt/C1	1.06	5.67	2.87	0.34
Pt/AC1	1.15	4.02	-	-
Pt/C2	-	1.30	3.76	0.49
Pt/AC3	1.15	2.14	0.75	0.33
Pt/AC4	3.11	1.73	0.78	0.09
Pt/AC4-1173	2.87	1.81	-	-
Pt/AC5	0.53	1.93	-	-

Supplementary Table 11. Acetylene adsorption capacities of fresh Pt/C catalysts determined via C_2H_2-TPD-MS and volumetric chemisorption.

Catalyst	TPD-MS			Volumetric chemisorption			
	$c_{C_2H_2}$[a] [μmol g^{-1}]	$c_{C_2H_2}$[b] [μmol g^{-1}]	T_{des}[c] [K]	$V_{phys,303K}$[d] [cm^3 g^{-1}]	$V_{chem,303K}$[e] [cm^3 g^{-1}]	$V_{phys,473K}$[f] [cm^3 g^{-1}]	$V_{chem,473K}$[g] [cm^3 g^{-1}]
Pt/AC	633	68	422	30.9	1.2	0.2	150.1
Pt/C1	1674	23	378	11.3	13.4	0	2.8
Pt/AC1	1189	54	388	38.9	1.9	0	25.3
Pt/C2	674	85	380	12.3	11.1	0	2.3
Pt/AC2	385	36	394	37.6	1.3	0.1	57.0
Pt/AC3	632	30	413	12.6	0.2	0.9	150.3
Pt/AC4	512	47	403	18.3	0.9	2.7	380.5
Pt/AC4-573	511	18	397	-	-	-	-
Pt/AC4-873	481	12	398	-	-	-	-
Pt/AC4-1173	536	68	410	24.5	0.8	0	289.3
Pt/AC5	571	54	418	24.1	0.9	0	188.8

[a]Total acetylene adsorption capacity, derived by integration of the whole data range between 303 K and 533 K. [b]Acetylene adsorption capacity above 473 K, derived by integration of the fraction of the data range between 473 K and 533 K. [c]Acetylene desorption temperature. [d]Volume of acetylene physisorbed at 303 K. [e]Volume of acetylene chemisorbed at 303 K. [f]Volume of acetylene physisorbed at 473 K. [g]Volume of acetylene chemisorbed at 473 K.

Supplementary Table 12. Acetylene adsorption capacities of fresh carbon supports and Ru/C and Au/C catalysts determined via volumetric chemisorption at 473 K.

Sample	$V_{phys,473K}$[a] [$cm^3\ g^{-1}$]	$V_{chem,473K}$[b] [$cm^3\ g^{-1}$]
AC	0.4	0.2
C1	0.4	0.2
AC3	0.5	0.1
AC4	0.2	0.4
AC5	0.3	0.1
Ru/AC	1.2	34.5
Ru/C1	0.9	2.8
Ru/AC4	0.0	33.4
Ru/AC5	0.8	29.0
Au/C1	0.9	2.6
Au/AC	0.0	16.8
Au/AC3	0.0	9.5
Au/AC5	0.0	24.6

[a]Volume of acetylene physisorbed. [b]Volume of acetylene chemisorbed.

Supplementary Table 13. Deactivation constants of Pt/C, Au/C, and Ru/C catalysts in acetylene hydrochlorination.

Catalyst	$k_{d,3h}$[a] $[h^{-1}]$	$k_{d,12h}$[b] $[h^{-1}]$
Pt/AC	-2.9	-1.3
Pt/AC1	-6.8	-2.4
Pt/AC2	-10.6	-1.6
Pt/AC3	0.0	0.0
Pt/AC4	-3.5	-1.3
Pt/AC4-573	-3.4	-1.0
Pt/AC4-873	0.0	-0.1
Pt/AC4-1173	-2.1	-0.9
Pt/AC5	0.0	-0.3
Ru/AC	-8.2	-2.2
Ru/AC4	-9.7	-2.5
Ru/AC5	-0.7	-0.9
Au/AC	-0.6	-0.3
Au/AC3	-0.7	-0.2
Au/AC5	-0.1	-0.1

[a,b]Linear regression in the data range of the first 3 and 12 h time-on-stream.

Supplementary Table 14. Fitting parameters derived from the Ru 3*p* XPS spectra of Ru/C catalysts.

Catalyst	Ru(0)[a]			RuO_2[a]			$RuCl_3$[a]		
	Position [eV]	FWHM[b] [eV]	Area [%]	Position [eV]	FWHM[b] [eV]	Area [%]	Position [eV]	FWHM[b] [eV]	Area [%]
Ru/AC	461.4	2.0	10	462.7	1.5	32	464.4	2.5	58
Ru/C1	461.4	2.0	28	462.7	1.5	24	464.4	2.5	48
Ru/AC4	461.2	2.0	10	462.7	1.9	27	464.4	2.5	63
Ru/AC5	461.4	1.4	14	462.8	1.5	30	464.4	2.5	56

[a]Assigned based on reference values.[3,11] [b]FWHM: full width at half maximum.

Supplementary Table 15. Results for mass and heat transfer limitation criteria.

Catalyst	$r_{v,obs}$ [$mol_{C_2H_2}$ s^{-1} m_{cat}^{-3}]	r_{pore}[a] [nm]	Ca[b] [-]	ΔT_e[c] [K]	Φ[d] [-]	ΔT_i[e] [K]
Pt/AC	3.7	1.2	0.0018	0.006	0.91	0.08
Pt/AC1	2.0	1.6	0.0009	0.004	0.36	0.11
Pt/AC2	1.9	1.5	0.0009	0.003	0.36	0.10

[a]Pore radius, estimated via nonlocal density functional theory. [b]Carberry criterion. [c]Extra-particle temperature gradient. [d]Weisz-Prater criterion. [e]Intra-particle temperature gradient.

Supplementary Figures

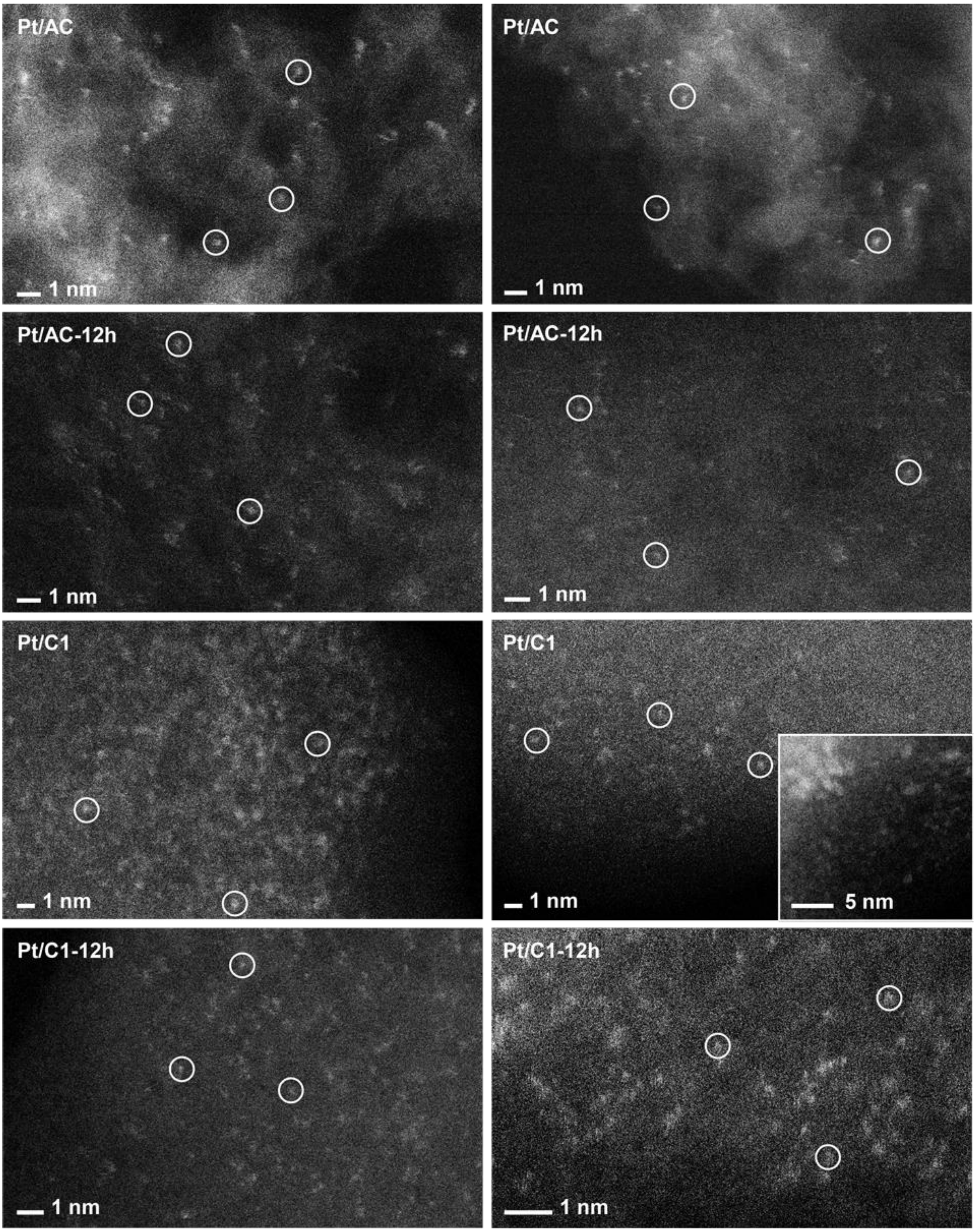

Supplementary Fig. 1. STEM of fresh and used Pt/AC and Pt/C1. Besides the predominant single atoms (examples highlighted by white circles), occasionally clusters and small nanoparticles were identified on Pt/C1 (inset).

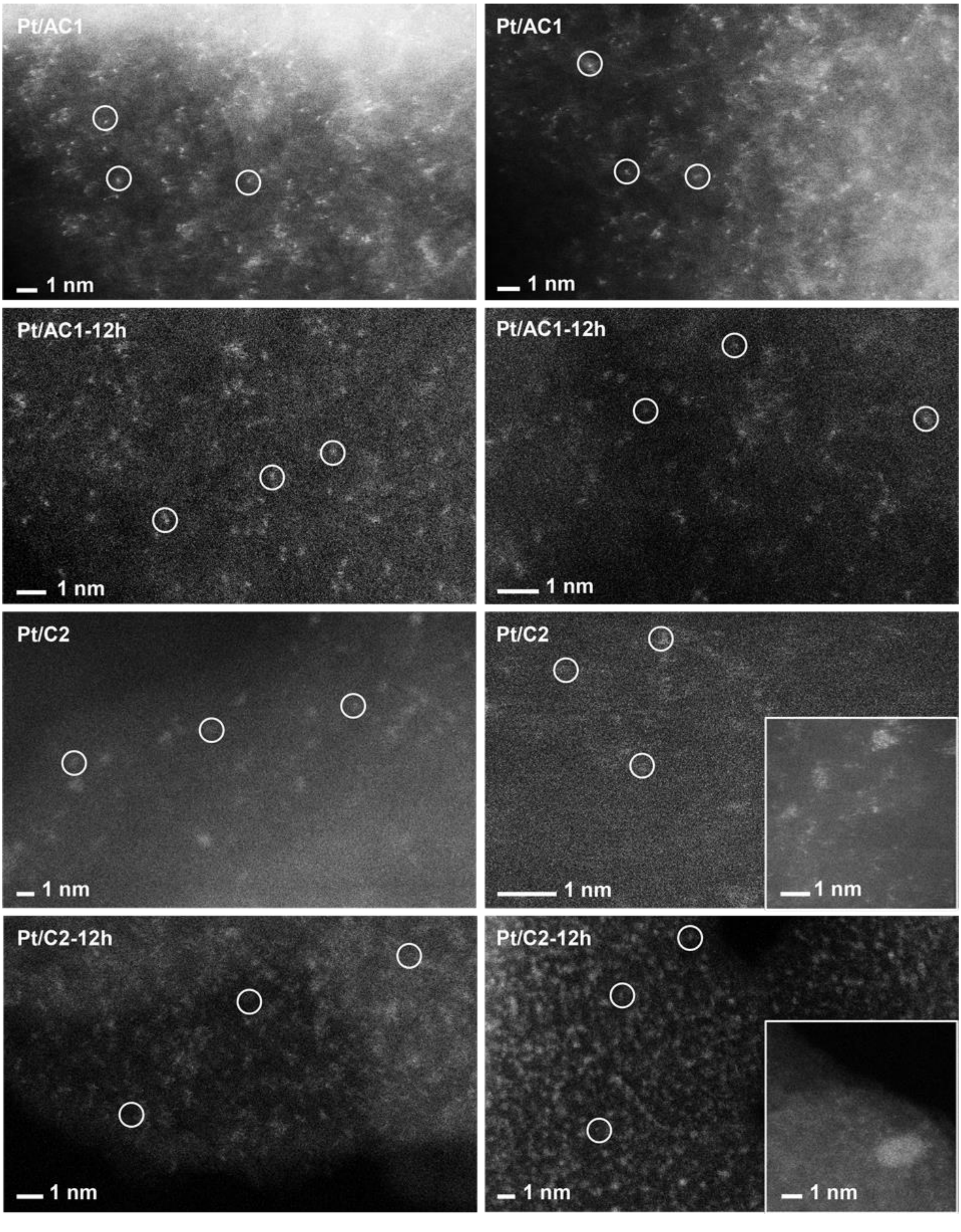

Supplementary Fig. 2. STEM of fresh and used Pt/AC1 and Pt/C2. Besides the predominant single atoms (examples highlighted by white circles), occasionally clusters and small nanoparticles were identified on Pt/C2 and Pt/C2-12h (insets).

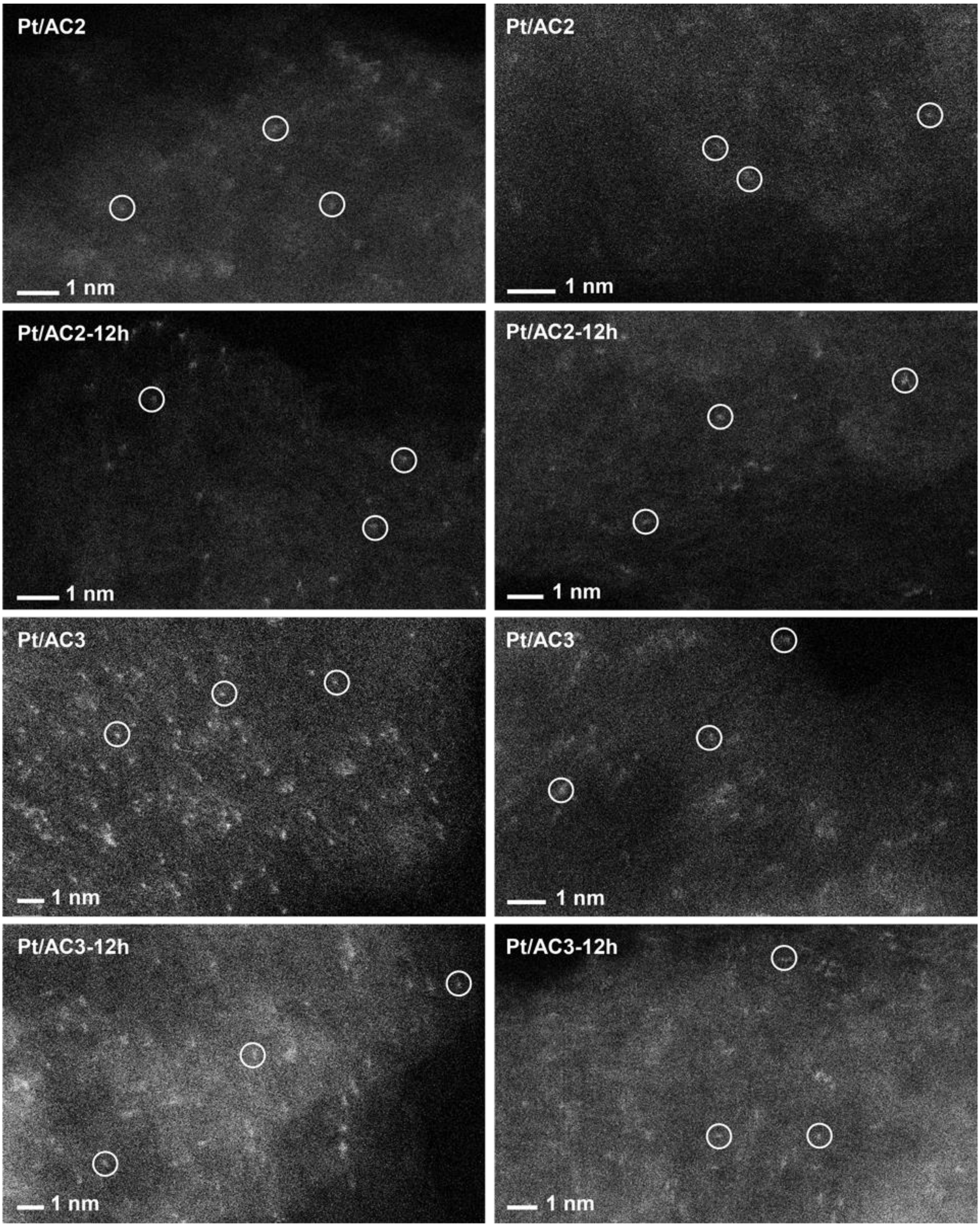

Supplementary Fig. 3. STEM of fresh and used Pt/AC2 and Pt/AC3, visualizing the exclusive presence of single atoms (examples highlighted by white circles).

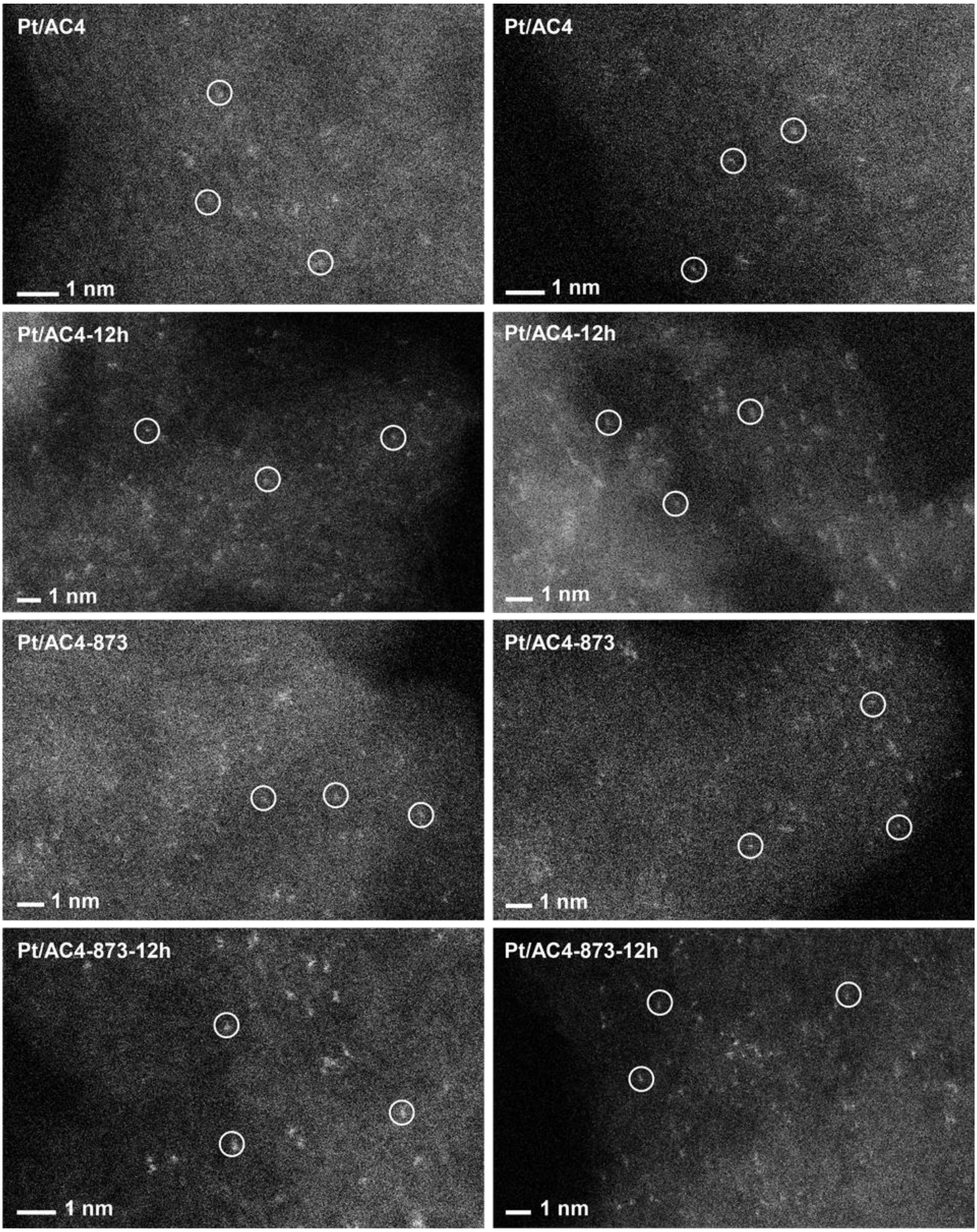

Supplementary Fig. 4. STEM of fresh and used Pt/AC4 and Pt/AC4-873, visualizing the exclusive presence of single atoms (examples highlighted by white circles).

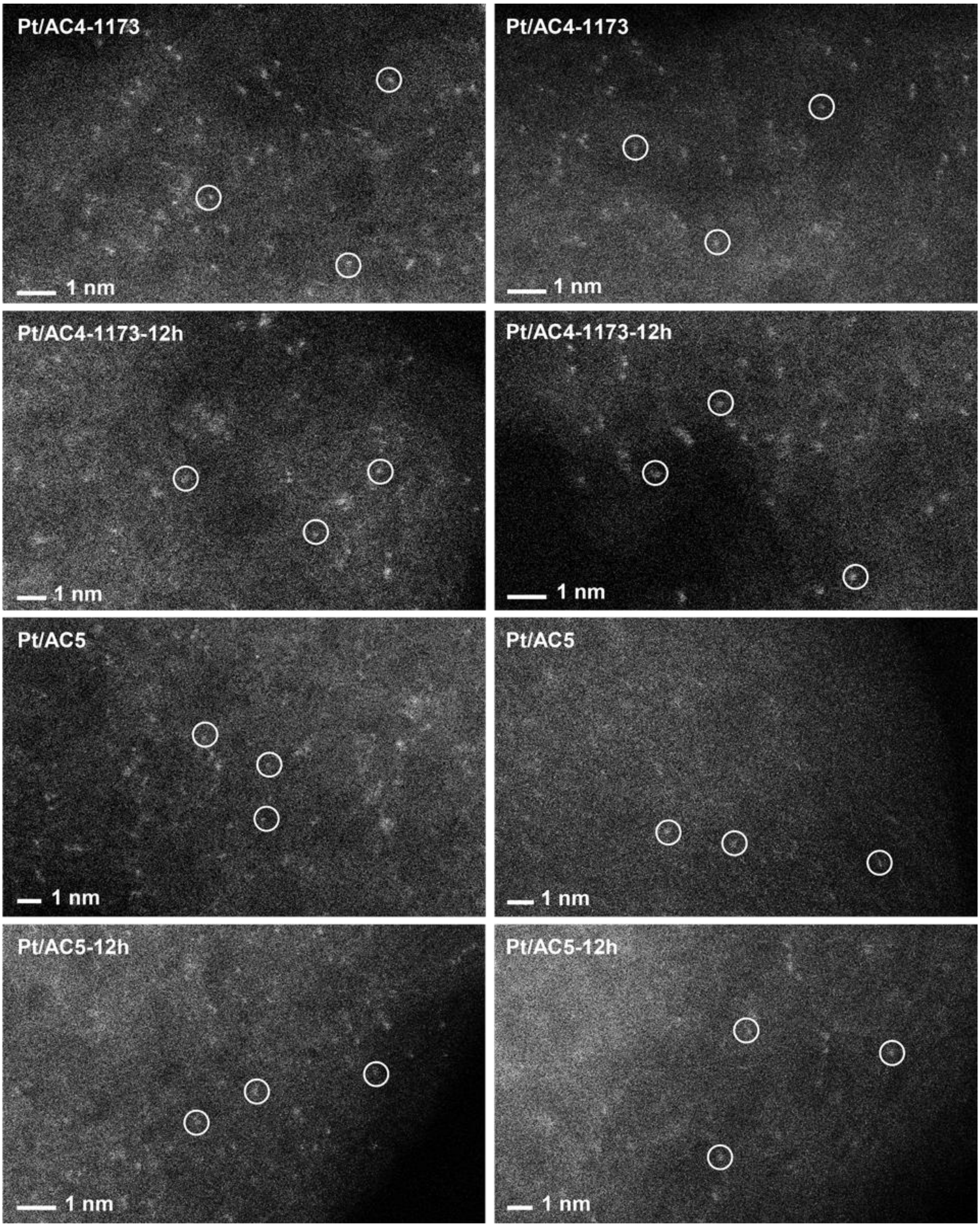

Supplementary Fig. 5. STEM of fresh and used Pt/AC4-1173 and Pt/AC5, visualizing the exclusive presence of single atoms (examples highlighted by white circles).

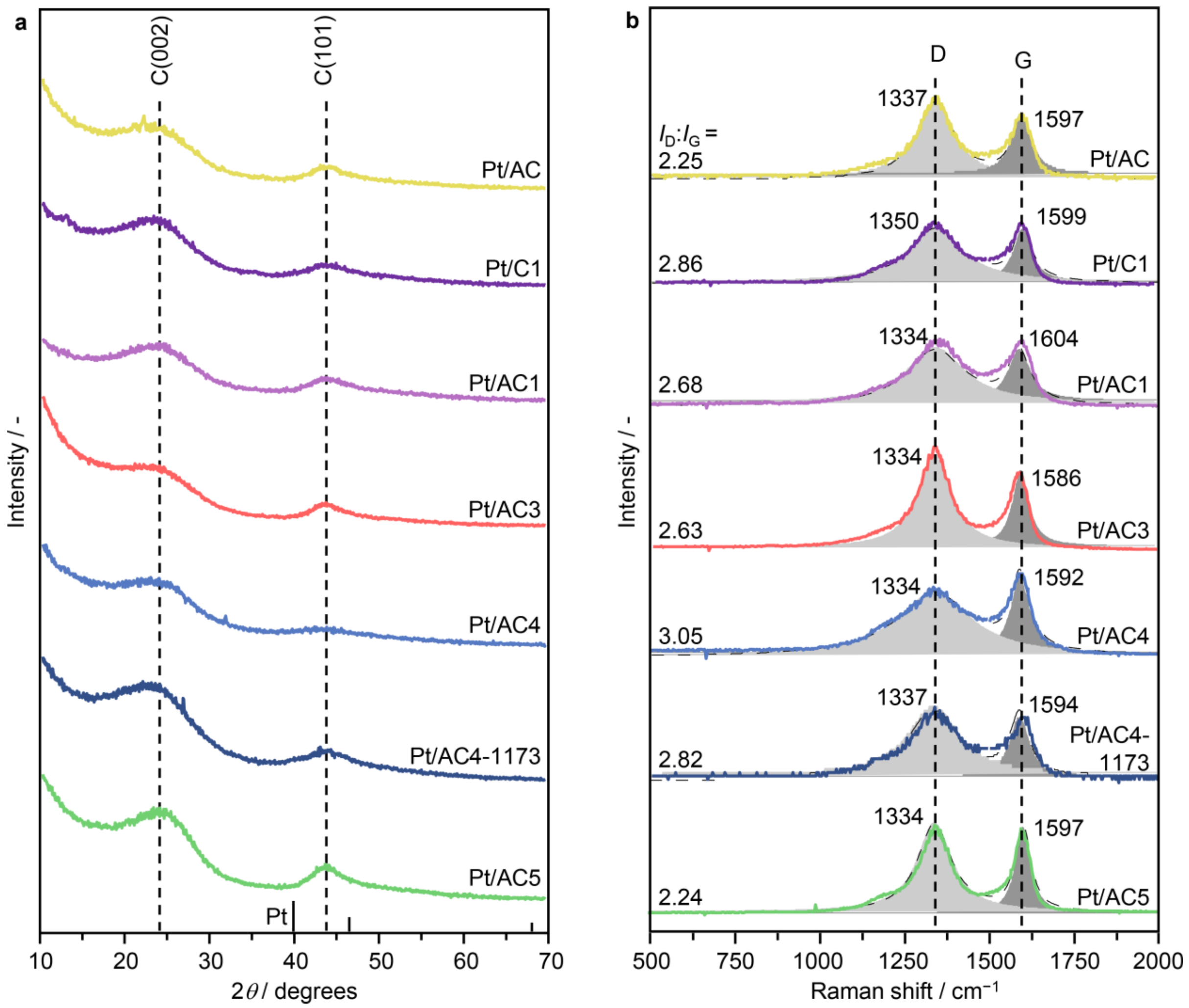

Supplementary Fig. 6. a, XRD pattern and **b,** Raman spectra of Pt/C catalysts. Diffraction peaks of Pt and carbon are indicated by vertical black bars and dashed black lines, respectively. Broad reflections at 24° and 44° 2θ suggest that all carbon supports are amorphous, which is in agreement with the Raman spectra, showing very broad D and G line features and a characteristic ratio >2.[22]

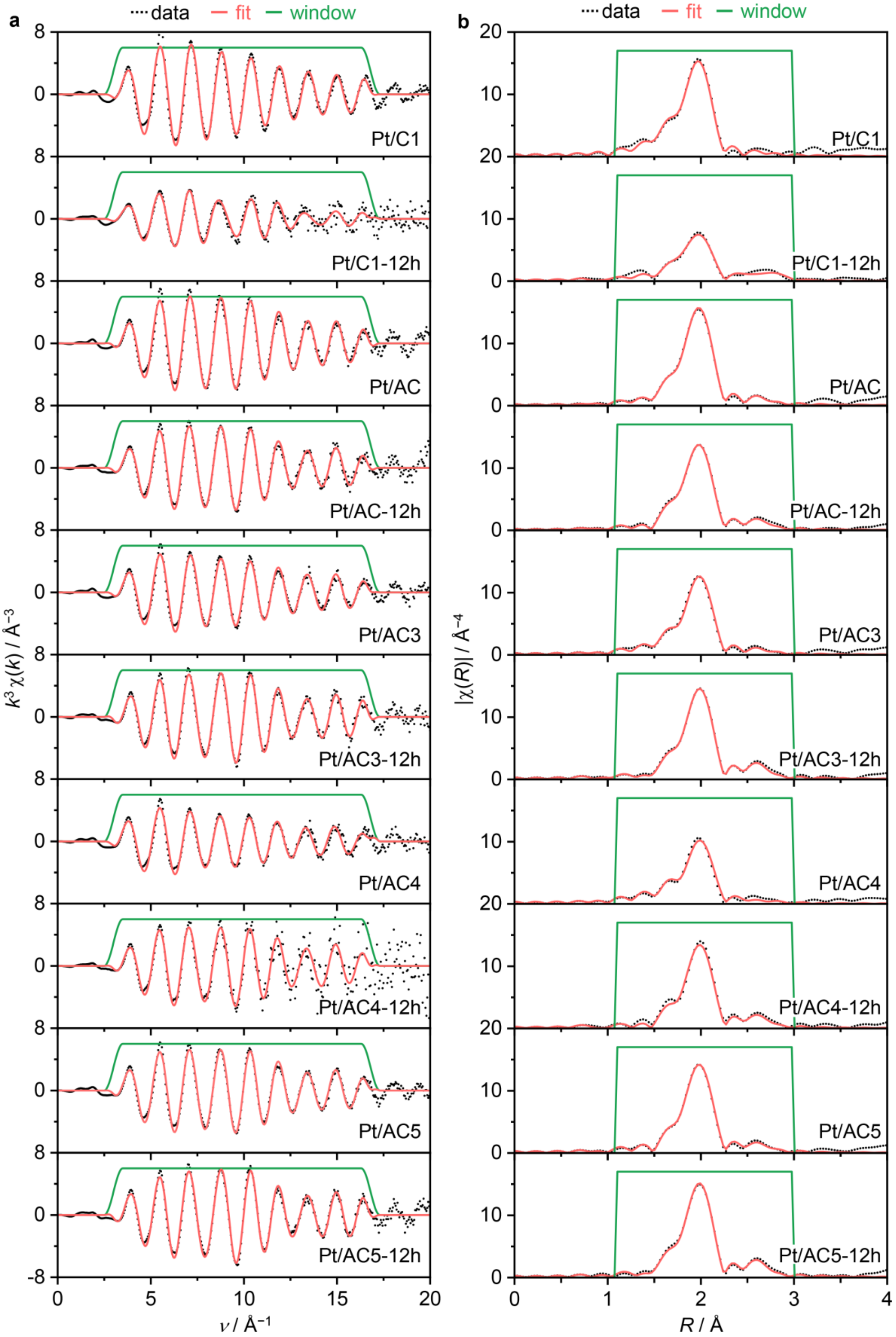

Supplementary Fig. 7. Experimental and fitted EXAFS spectra at the Pt L_3 edge of fresh and used Pt/C catalysts. **a,** *k*-space. **b,** *R*-space.

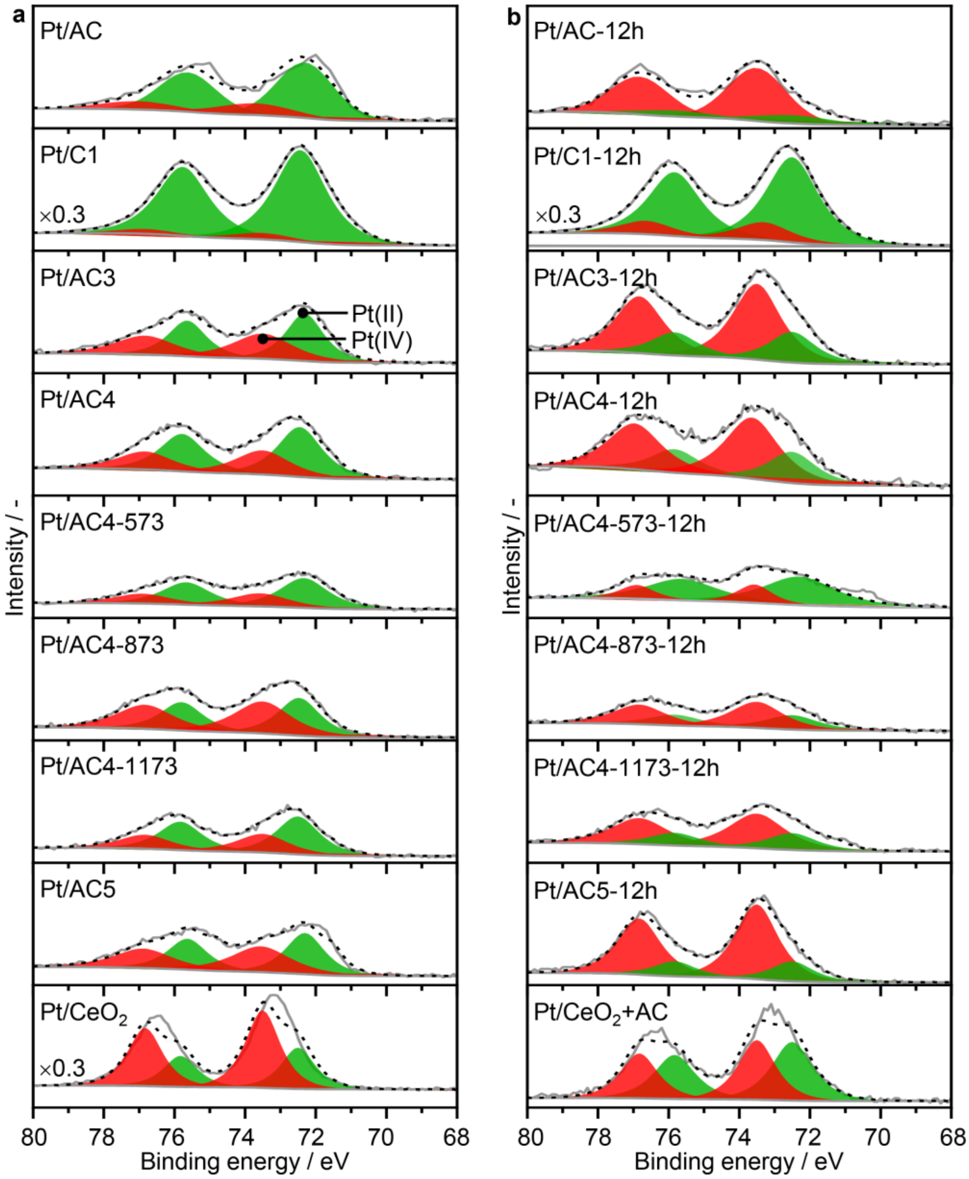

Supplementary Fig. 8. Pt 4*f* XPS spectra of **a,** fresh Pt/C and Pt/CeO_2 catalysts and **b,** after use in acetylene hydrochlorination or after ball milling with AC (Pt/CeO_2+AC).

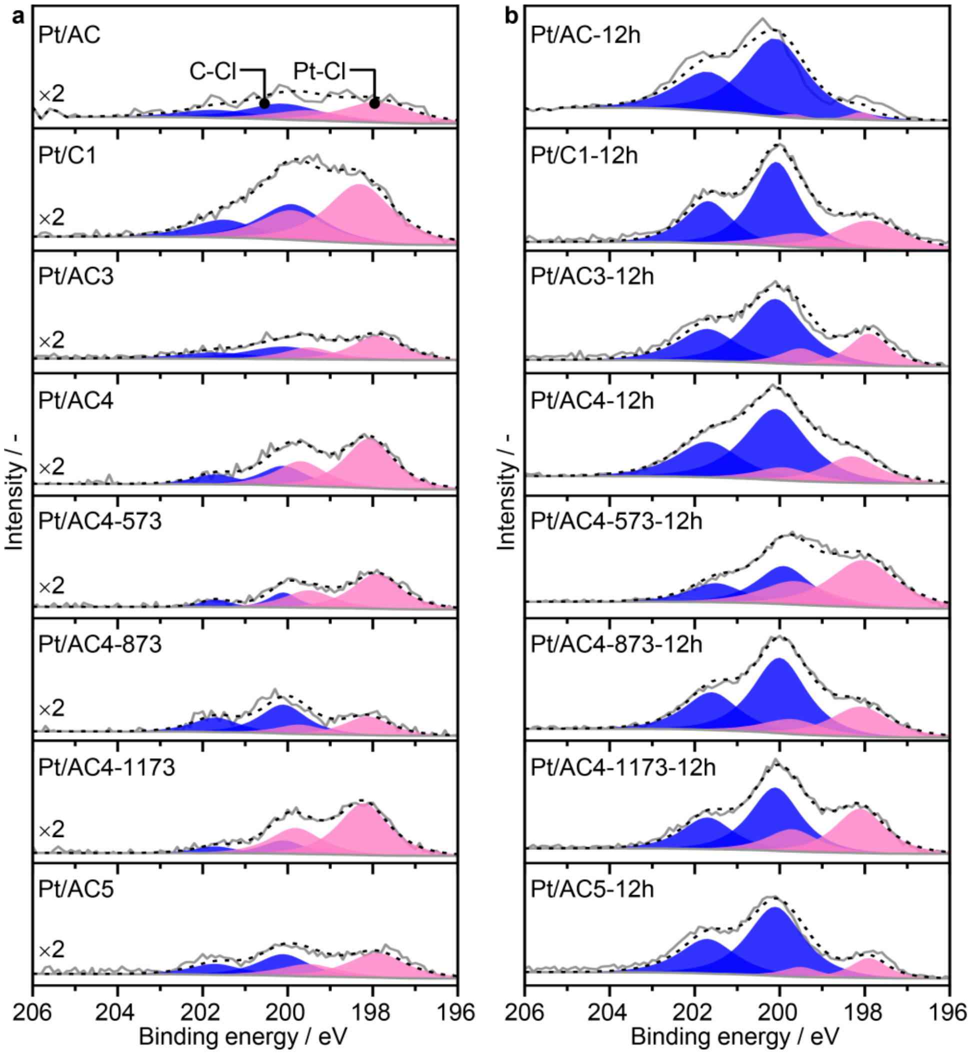

Supplementary Fig. 9. Cl 2*p* XPS spectra of **a,** fresh and **b,** used Pt/C catalysts.

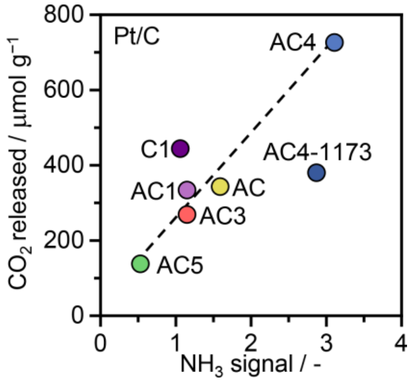

Supplementary Fig. 10. Correlation between acidic sites, as probed by NH_3-TPD-MS and the amount of CO_2 released during TGA-MS in flowing He of the fresh Pt/C catalysts.

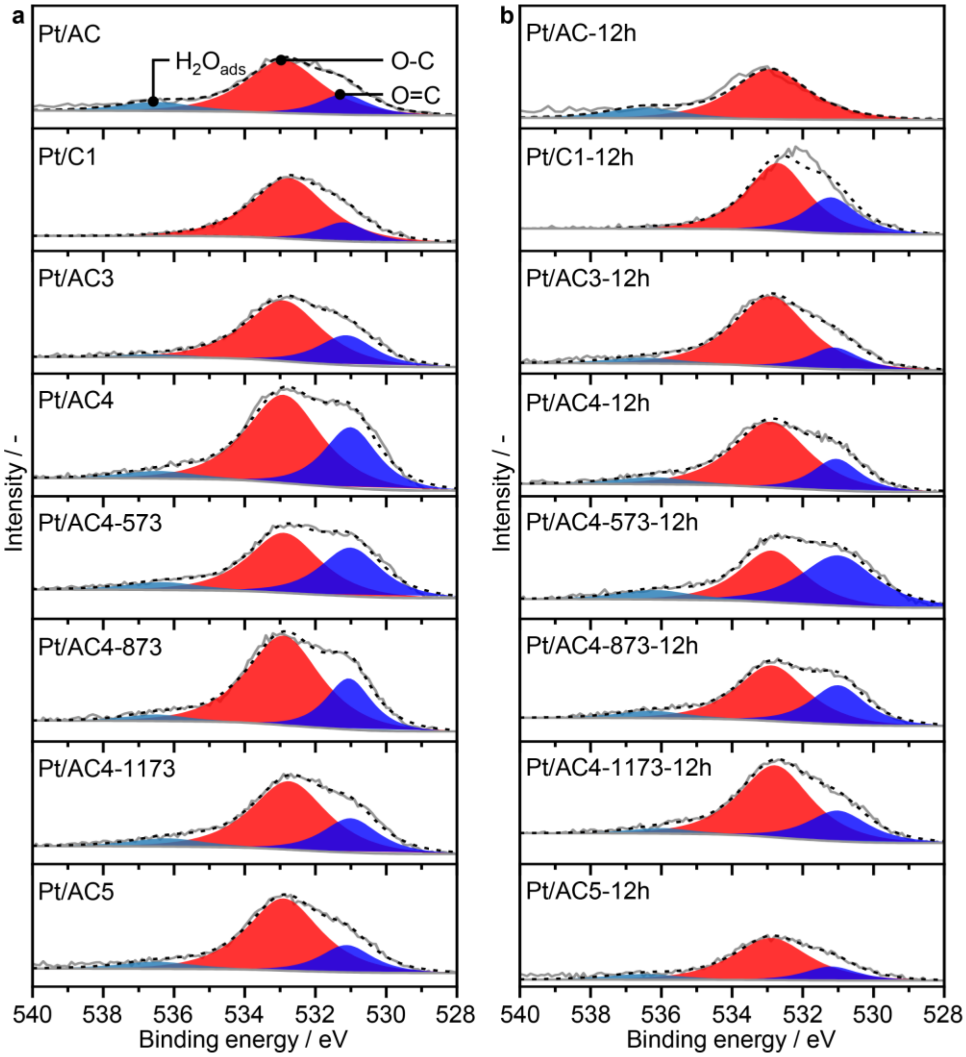

Supplementary Fig. 11. O 1*s* XPS spectra of **a,** fresh and **b,** used Pt/C catalysts.

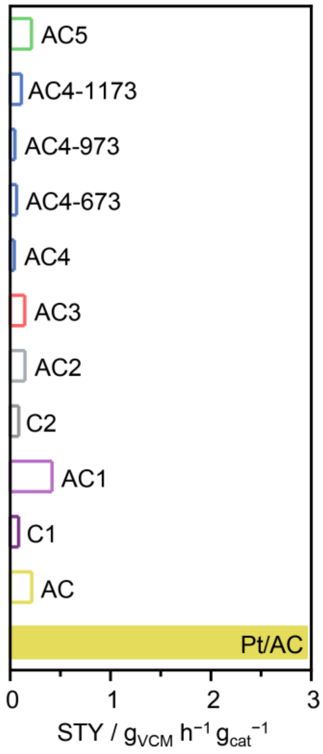

Supplementary Fig. 12. Initial activity of the pure carbon supports in acetylene hydrochlorination and the comparison to Pt/AC. Reaction conditions: T_{bed} = 473 K, HCl:C_2H_2 = 1.1:1, GHSV(C_2H_2) = 1500 h^{-1}.

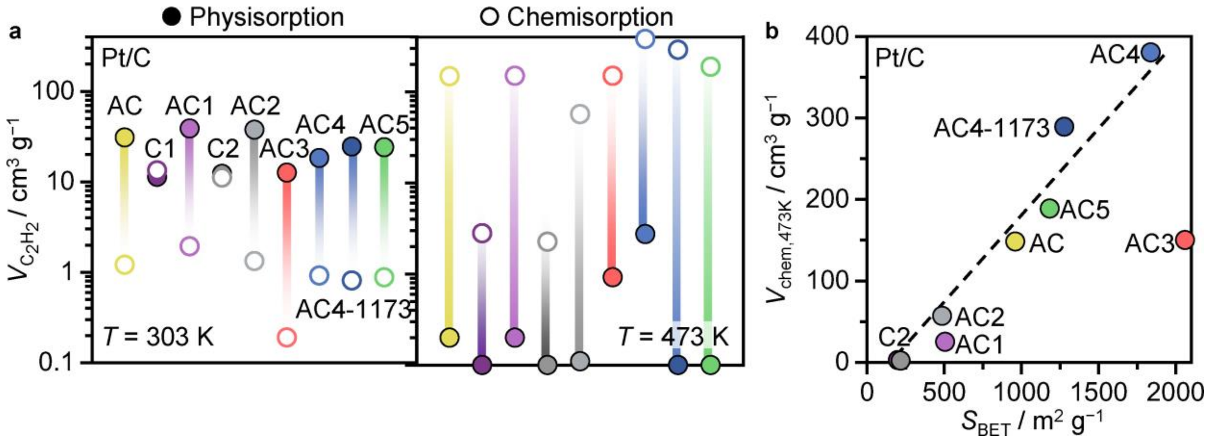

Supplementary Fig. 13. a, Acetylene adsorption capacity determined by volumetric chemisorption at 303 K and 473 K. **b,** Correlation between acetylene adsorption capacity and surface area of Pt/C catalysts.

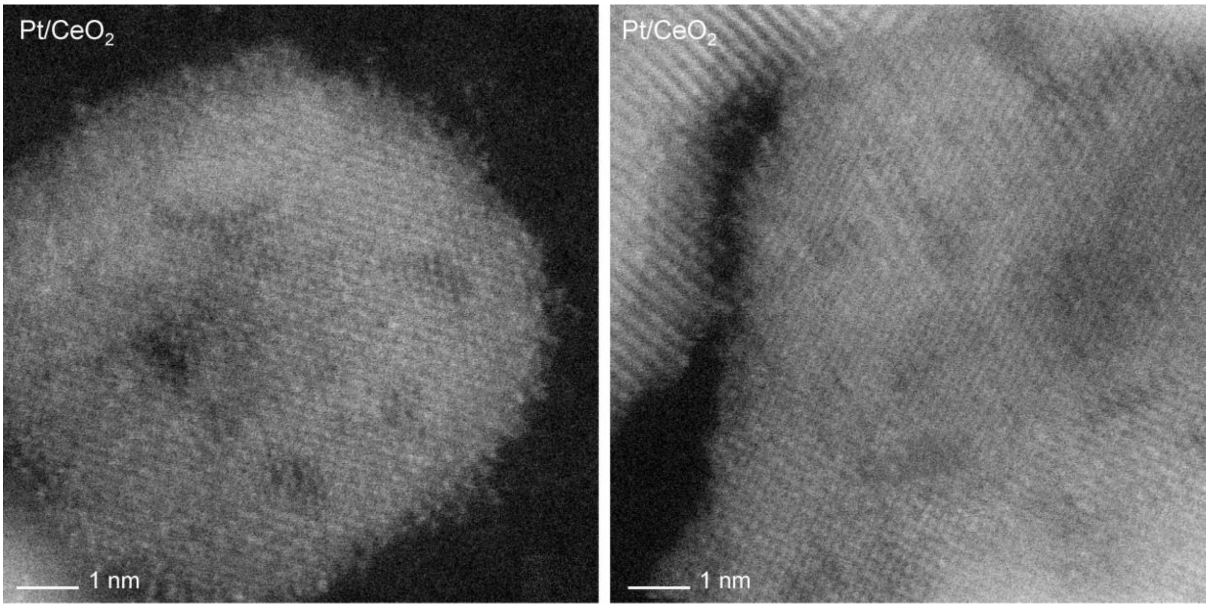

Supplementary Fig. 14. STEM of Pt/CeO_2, visualizing Pt single atoms.

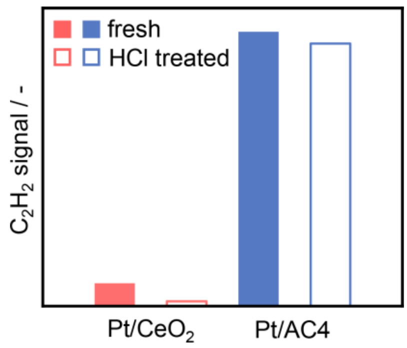

Supplementary Fig. 15. Acetylene adsorption capacity of Pt/CeO_2 and Pt/AC4 before and after exposure to HCl (T = 473 K, t = 30 min, flowing HCl). While the pre-treatment only marginally affects the acetylene interaction with the carbon-based catalyst, the ability of the ceria-based catalyst to adsorb acetylene vanishes, as a consequence of the extensive chlorination of the support.[23]

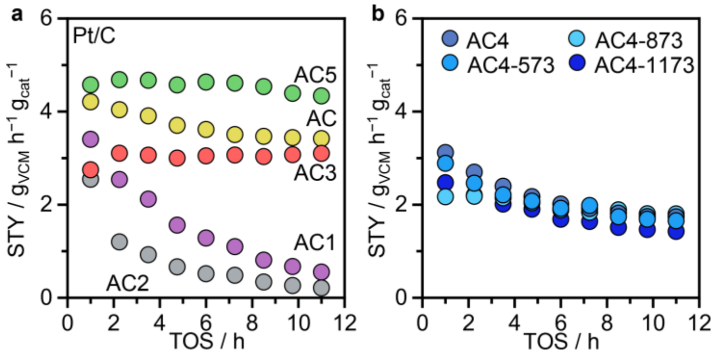

Supplementary Fig. 16. a,b, Stability tests of Pt/C catalysts. The corresponding deactivation constants are given in **Supplementary Table 13.** Reaction conditions: T_{bed} = 473 K, HCl:C_2H_2 = 1.1:1, GHSV(C_2H_2) = 650 h^{-1}.

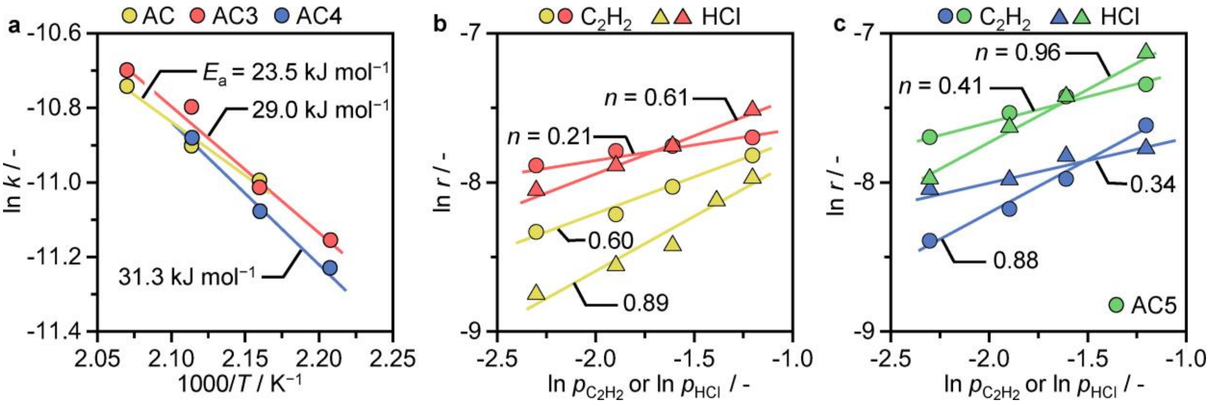

Supplementary Fig. 17. Kinetics of acetylene hydrochlorination on selected Pt/C catalysts. **a,** Arrhenius plots used to derive the apparent activation energy, E_a. **b,c,** Reaction rate, *r*, as a function of the inlet partial pressure of C_2H_2 or HCl. The partial reaction order of both reactants, *n*, is indicated by the slope of the fitting lines. Reaction conditions: $T_{bed} = 473$ K, $F_T = 20$ cm^3 min^{-1}, $W_{cat} = 0.1$ g, and $P = 1$ bar. In order to circumvent the influence of catalyst deactivation, each point was obtained in a single experiment, after 15 min TOS.

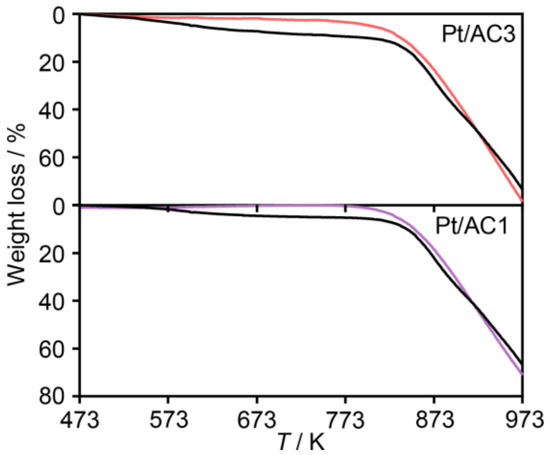

Supplementary Fig. 18. Thermogravimetric analysis in 20 vol.% O_2/Ar of fresh (colored profiles) and used Pt/C catalysts (black profiles). The difference in weight loss between the fresh and used samples suggests a comparable amount of coke deposits in the two catalysts, estimated at *ca.* 4 wt.%, which is well in line with their similar content of acidic oxygen functionalities.

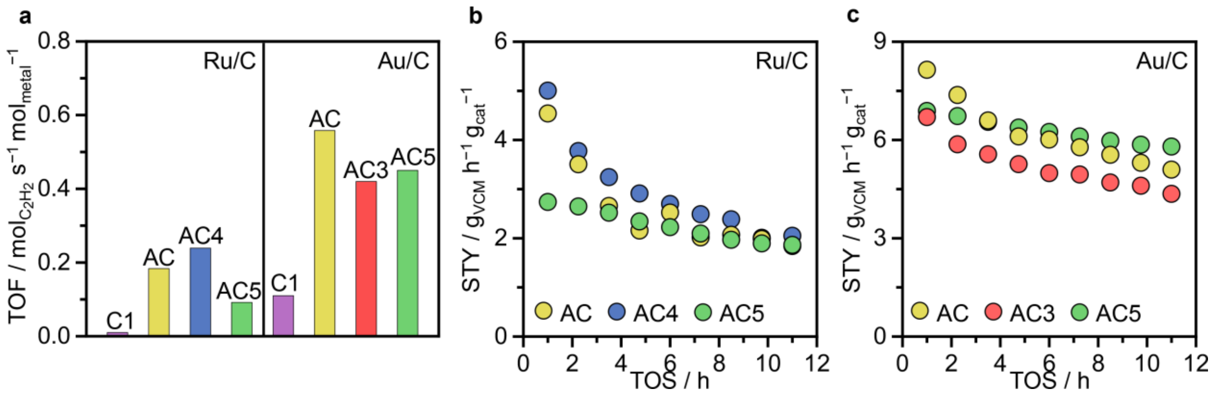

Supplementary Fig. 19. a, Initial activity and **b,c,** stability tests of Ru/C and Au/C catalysts in acetylene hydrochlorination. The corresponding deactivation constants are given in **Supplementary Table 13.** Reaction conditions: T_{bed} = 473 K, HCl:C_2H_2 = 1.1:1, GHSV(C_2H_2) = 650 h^{-1}.

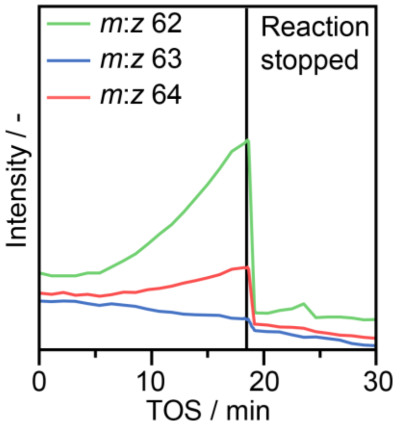

Supplementary Fig. 20. Evolution of the *m*:*z* 62, *m*:*z* 63, *m*:*z* 64 ions over ^{13}C-labeled N-doped carbon as a function of time-on-stream in acetylene hydrochlorination as determined by MS analysis. The solid black line indicates the moment when the C_2H_2 and HCl feed was stopped.

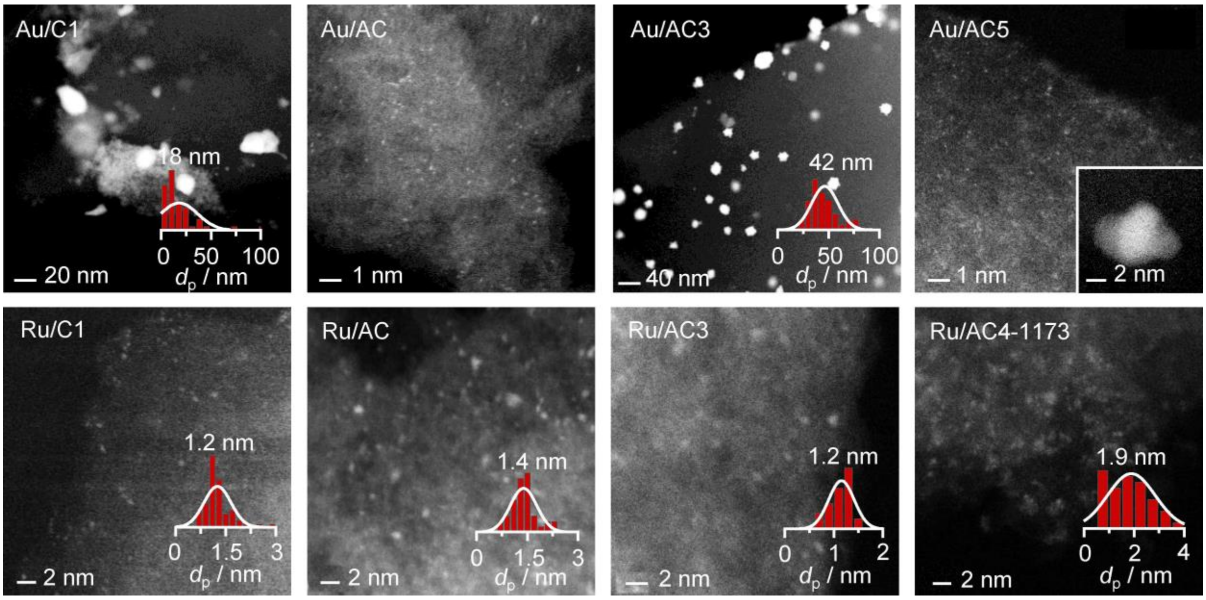

Supplementary Fig. 21. STEM of fresh Au/C and Ru/C catalysts. The gold nanostructure strongly depends on the choice of the carbon host and varies from large nanoparticles (C1) to single atoms (AC). In the case of ruthenium, small nanoparticles were obtained, regardless of the type of carbon support. Particle size distributions were derived from analysis of >100 particles.

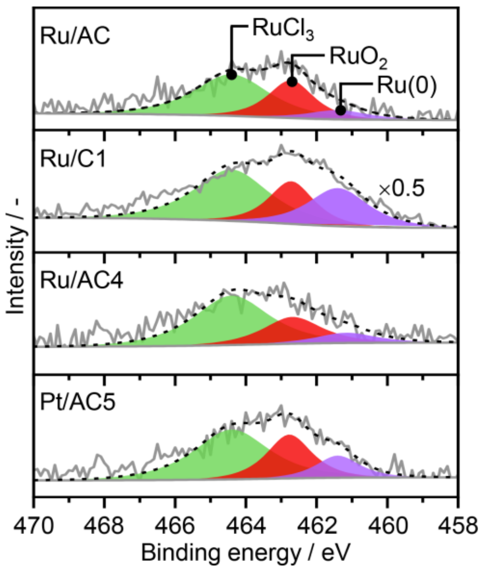

Supplementary Fig. 22. Ru 3*p* XPS spectra of fresh Ru/C catalysts.

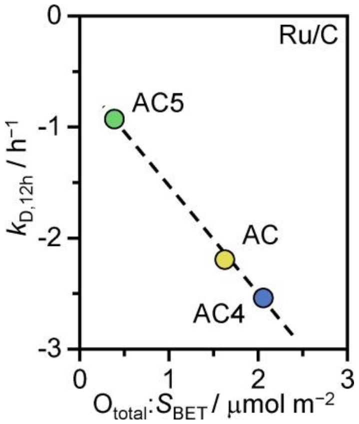

Supplementary Fig. 23. Correlation between the deactivation constant of Ru/C catalysts in acetylene hydrochlorination and the density of surface oxygen functionalities in the fresh support, estimated as the total content of oxygen functional groups evolving as CO_2 and CO during TPD-MS normalized by the surface area.

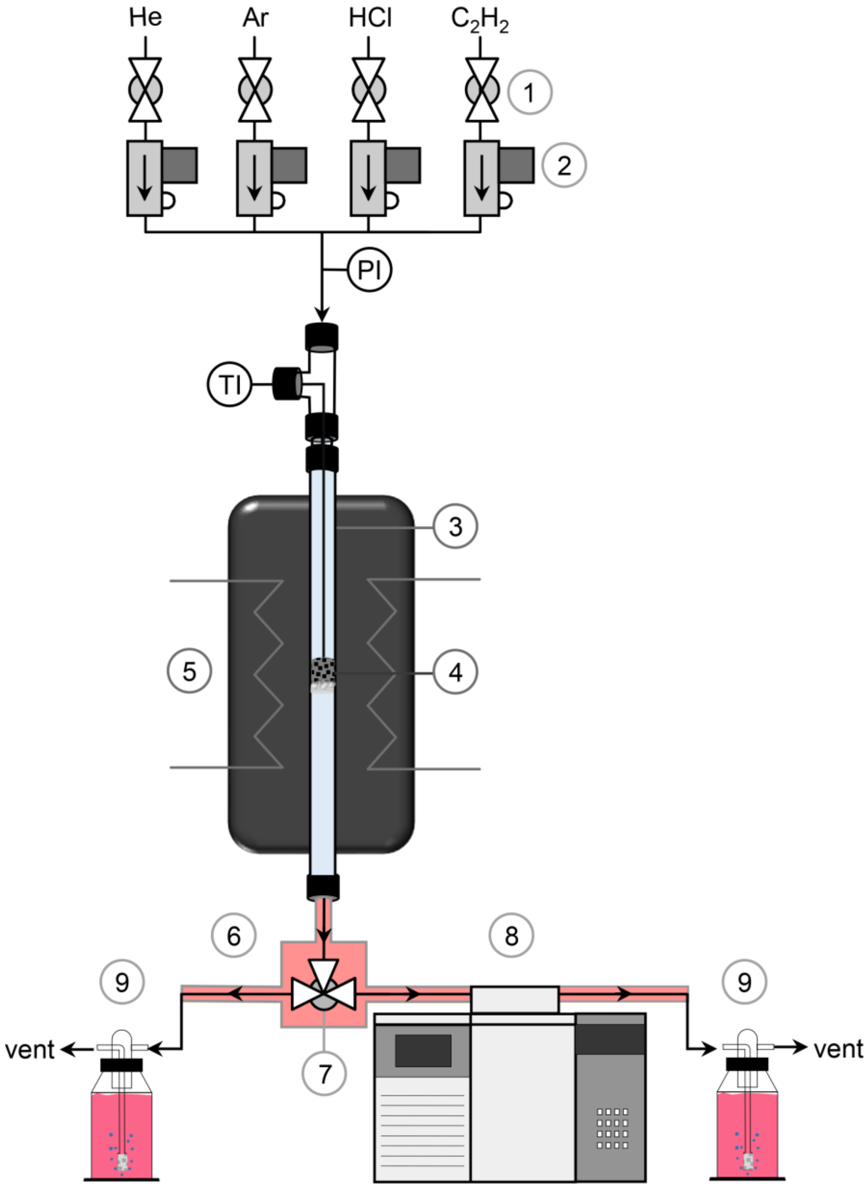

Supplementary Fig. 24. Scheme of the laboratory set-up used for acetylene hydrochlorination. 1: two-way on-off valves, 2: mass-flow controllers, 3: quartz reactor, 4: catalyst bed, 5: oven, 6: heat tracing (red coloring), 7: three-way sampling valve, 8: gas chromatograph connected to a mass spectrometer, 9: NaOH scrubbers, PI: pressure indicator, and TI: temperature indicator.

Supplementary References

1 Lin, R., Kaiser, S. K., Hauert, R. & Pérez-Ramírez, J. Descriptors for high-performance nitrogen-doped carbon catalysts in acetylene hydrochlorination. *ACS Catal.* **8**, 1114-1121 (2018).

2 Kaiser, S. K. et al. Nanostructuring unlocks high performance of platinum single-atom catalysts for stable vinyl chloride production. *Nat. Catal.* **3**, 376-385 (2020).

3 Kaiser, S. K. et al. Preserved in a shell: high-performance graphene-confined ruthenium nanoparticles in acetylene hydrochlorination. *Angew. Chem. Int. Ed.* **58**, 12297-12304 (2019).

4 Sun, X. et al. Facile synthesis of precious-metal single-site catalysts using organic solvents. *Nat. Chem.* **12**, 560-567 (2020).

5 Pereira-Hernández, X. I. et al. Tuning Pt-CeO_2 interactions by high-temperature vapor-phase synthesis for improved reducibility of lattice oxygen. *Nat. Commun.* **10**, 1358 (2019).

6 Muller, O. et al. Quick-EXAFS setup at the SuperXAS beamline for *in situ* X-ray absorption spectroscopy with 10 ms time resolution. *J. Synchrotron Radiat.* **23**, 260-266 (2016).

7 Clark, A. H., Imbao, J., Frahm, R. & Nachtegaal, M. ProQEXAFS: A highly optimized parallelized rapid processing software for QEXAFS data. *J. Synchrotron Radiat.* **27**, 551-557 (2020).

8 Ravel, B. & Newville, M. ATHENA, ARTEMIS, HEPHAESTUS: data analysis for X-ray absorption spectroscopy using IFEFFIT. *J. Synchrotron Radiat.* **12**, 537-541 (2005).

9 Moulder, J. F., Stickle, W. F., Sobol, P. E. & Bomben, K. D. *Handbook of X-Ray Photoelectron Spectroscopy* 62-63 (Physical Electronics, Inc., 1995).

10 Zhou, J.-H. et al. Characterization of surface oxygen complexes on carbon nanofibers by TPD, XPS and FT-IR. *Carbon* **45**, 785-796 (2007).

11 Morgan, D. J. Resolving ruthenium: XPS studies of common ruthenium materials. *Surf. Interface Anal.* **47**, 1072-1079 (2015).

12 Carberry, J. J. in *Catalysis: Science and Technology* Ch. 3, 131-171 (Springer-Verlag, 1987).

13 Mears, D. Diagnostic criteria for heat transport limitations in fixed bed reactors. *J. Catal.* **20**, 127-131 (1971).

14 Weisz, P. B. & Prater, C. D. Interpretation of measurements in experimental catalysis. *Adv. Catal.* **6**, 143-196 (1954).

15 Yang, M. et al. A common single-site Pt(II)-$O(OH)_x$- species stabilized by sodium on "active" and "inert" supports catalyzes the water-gas shift reaction. *J. Am. Chem. Soc.* **137**, 3470-3473 (2015).

16 Kaiser, S. K. et al. Single-atom catalysts across the periodic table. *Chem. Rev.* **120**, 11703-11809 (2020).

17 Cazorla-Amorós, D., Alcañiz-Monge, J., de la Casa-Lillo, M. A. & Linares-Solano, A. CO_2 as an adsorptive to characterize carbon molecular sieves and activated carbons. *Langmuir* **14**, 4589-4596 (1998).

18 Lozano-Castelló, D., Cazorla-Amorós, D. & Linares-Solano, A. Usefulness of CO_2 adsorption at 273 K for the characterization of porous carbons. *Carbon* **42**, 1233-1242 (2004).

19 Linares-Solano, A., Salinas-Marítnez de Lecea, C., Alcañiz-Monge, J. & Cazorla-Amorós, D. Further advances in the characterization of microporous carbons by physical adsorption of gases. *Tanso* **1998**, 316-325 (1998).

20 Cazorla-Amorós, D., Alcañiz-Monge, J. & Linares-Solano, A. Characterization of activated carbon fibers by CO_2 adsorption. *Langmuir* **12**, 2820-2824 (1996).
21 G. Beamson, D. B. *High Resolution XPS of Organic Polymers, the Sienta ESCA300 Database*. Vol. 5 295 (Wiley, 1992).
22 Schwan, J. et al. Raman spectroscopy on amorphous carbon films. *J. Appl. Phys.* **80**, 440-447 (1996).
23 Amrute, A. P. et al. Performance, structure, and mechanism of CeO_2 in HCl oxidation to Cl_2. *J. Catal.* **286**, 287-297 (2012).
